# (Hetero)arene-fused boroles: a broad spectrum of applications

**DOI:** 10.1039/d0sc05676f

**Published:** 2020-11-24

**Authors:** Jiang He, Florian Rauch, Maik Finze, Todd B. Marder

**Affiliations:** Institute for Inorganic Chemistry and Institute for Sustainable Chemistry & Catalysis with Boron (ICB), Julius-Maximilians-Universität Würzburg Am Hubland 97074 Würzburg Germany maik.finze@uni-wuerzburg.de todd.marder@uni-wuerzburg.de

## Abstract

(Hetero)arene-fused boroles are a class of compounds containing a 5-membered boron diene-ring. Based on their molecular framework, the (hetero)arene-fused boroles can be considered as boron-doped polycyclic antiaromatic hydrocarbons and are thus of great interest. Due to the vacant p_*z*_ orbital on the 3-coordinate boron atom, the antiaromaticity and strain of the 5-membered borole ring, (hetero)arene-fused boroles possess strong electron accepting abilities and Lewis acidity. By functionalization, they can be tuned to optimize different properties for specific applications. Herein, we summarize synthetic methodologies, different strategies for their functionalization, and applications of (hetero)arene-fused boroles.

## Abstract 2

(Hetero)arene-fused boroles, ‘antiaromatic’ 2*n*-electron π-systems, more stable and more functionalizable than boroles, offer greater potential for a variety of applications.

## Graphical abstract

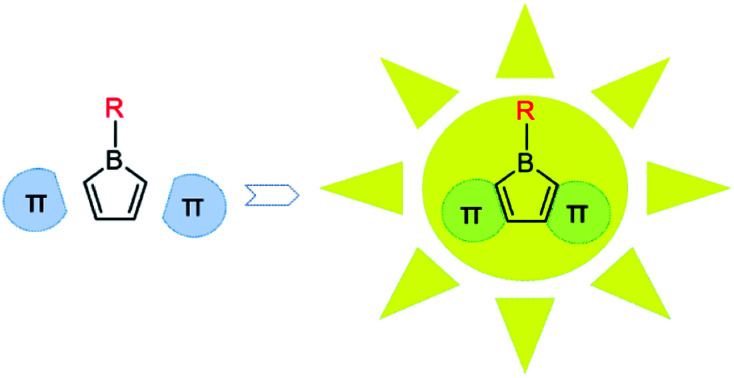

## Introduction

**Fig. 1:**
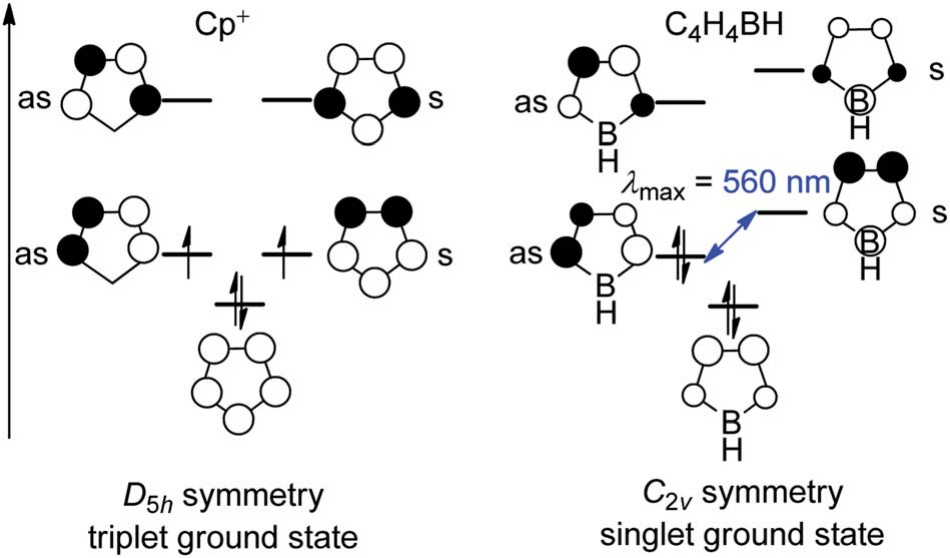
Molecule orbitals of the cyclopentadiene cation (Cp^+^) and borole; “as” and “s” denote the antisymmetric and symmetric orbitals, respectively, with respect to the mirror plane perpendicular to the molecule.

Three-coordinate boranes have been studied intensely for applications such as anion sensors,^1–3^ nonlinear optical materials (NLOs),^4–14^ live cell imaging,^15–18^ sensing of DNA, RNA and proteins,^19,20^*etc.*^21–32^ Among them, boroles are distinct, being 5-membered unsaturated 4π-electron heterocycles containing a 3-coordinate boron center. Interest in boroles originates from their being isoelectronic with the cyclopentadiene cation (Cp^+^) which, in terms of Hückel's rule,^33–36^ is antiaromatic and thus highly reactive. The isolation of “free” Cp^+^ has not been achieved. Cp^+^ has a triplet electronic ground state, which was confirmed by ESR spectroscopic measurements of the pentaphenylcyclopentadienyl cation at low temperature.^37^ The *C*_2v_ symmetry of a borole is lower than that of Cp^+^ (*D*_5h_, Fig. 1), which results in a splitting of the previously degenerate half-filled molecular orbitals. The orbital with a nodal plane passing through the boron atom (“as” in Fig. 1) is lowered in energy and occupied, which leads to a singlet ground state and diamagnetic character of boroles, in contrast to the biradical character of Cp^+^. The small HOMO–LUMO gap in boroles leads to their intense color.

Similar to Cp^+^, the “free” HBC_4_H_4_ is inaccessible due to its high reactivity caused by its antiaromaticity^38–40^ and low lying LUMO. *Via* steric protection, monomeric pentaphenylborole (PhBC_4_Ph_4_, PhB) was first synthesized in 1969 by Eisch and co-workers,^41^ but its crystal structure was only determined in 2008 by Braunschweig and co-workers.^42^ Steric protection of boroles was not only achieved with phenyl groups but also with other even bulkier protecting groups, *e.g.*, ^F^Ph (pentafluorophenyl),^43^ Mes (2,4,6-trimethylphenyl)^44^ and ^F^Mes (2,4,6-tritrifluoromethylphenyl).^45^ However, these non-fused “free” boroles are still highly reactive compounds. The chemistry of non-fused “free” boroles was previously reviewed by Eisch,^46^ Marder and co-workers,^47^ Braunschweig and co-workers,^48–50^ Wakamiya,^51^ Martin and co-workers^52^ and Kinjo and co-workers.^53,54^

**Scheme 1:**
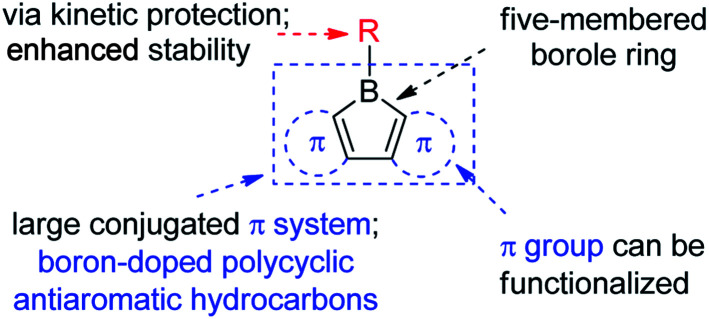
(Hetero)arene-fused boroles.

The stability of boroles is largely enhanced by annulation, and fused boroles are readily accessible and functionalizable (Scheme 1). In comparison to related triarylboranes, the electron accepting ability and Lewis acidity of the boron atom is largely enhanced in boroles, as a result of the antiaromaticity and strain of the 5-membered borole ring. Such fused boroles can be considered to be boron-doped polycyclic antiaromatic hydrocarbons.

The first dibenzo-fused borole, namely the parent 9-borafluorene (Bf), was reported in the early 1960s by Köster and Benedikt,^55^ but only at the start of this century, has the chemistry of Bfs started to attract increasing interest. (Hetero)arene-fused boroles have been reviewed very briefly either as part of reviews on “free” boroles^47,49,51,54^ or on boron-doped polycyclic aromatic hydrocarbons (PAHs)^56,57^ or in the context of subvalent boranes.^58^ In this review, we address the synthesis, properties and applications of (hetero)arene-fused boroles in detail, our primary focuses are: (i) the optoelectronic behavior of the compounds; and (ii) their classification based on structure property relationships. New developments in the use of the *exo*-substituents on boron are also discussed. The structures of all compounds with Arabic numerals are shown in Scheme 25 at the end of the paper for the reader's convenience.

## Synthetic methodology

There are three main approaches for the synthesis of 9-aryl-9-borafluorenes: (1) the use of easy-to-functionalize 9-X-9-borafluorenes (XBf, X = Cl or Br or I) as key intermediate; (2) assembling 9-aryl-9-borafluorenes in one step; and (3) stepwise substitution reactions at boron.

**Scheme 2:**
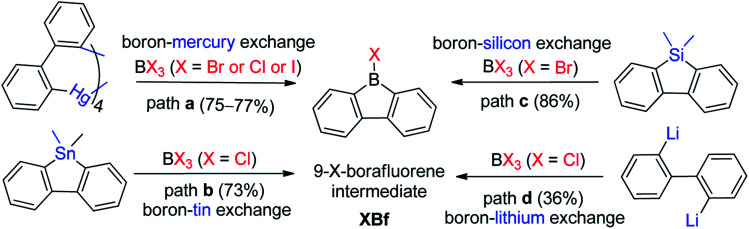
Synthesis of 9-X-9-borafluorenes.

The first approach relies on readily available 9-X-9-borafluorenes (XBf, X = Cl or Br or I). Four synthetic approaches to XBf derivatives have been developed. In 1985, Nöth and co-workers applied a boron–mercury exchange reaction (Scheme 2, path a).^59^ Analogous to the preparation of PhB,^60^ a boron–tin exchange reaction can also be utilized for the synthesis of XBfs (Scheme 2, path b).^61,62^ To avoid using these highly toxic metals, a boron–silicon exchange reaction was developed (Scheme 2, path c).^63^ClBf can be obtained directly from reaction of 2,2′-dilithiobiphenyl with BCl_3_ in an aliphatic solvent (Scheme 2, path d).^64^ The advantage of this approach is that once intermediate XBf is obtained, it is useful for subsequent derivatization.

**Scheme 3:**
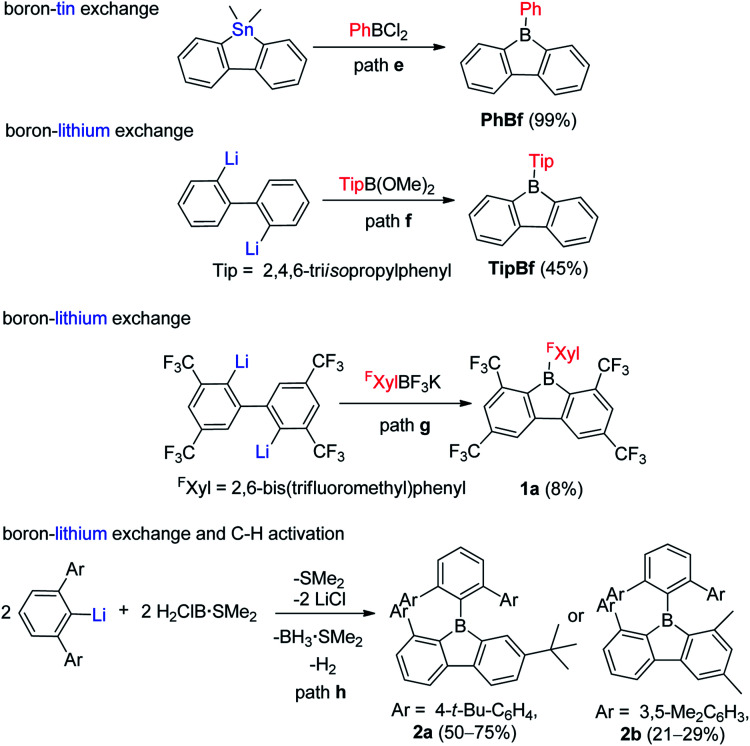
One-step synthesis of 9-aryl-9-borafluorenes.

For the one-step assembly of 9-aryl-9-borafluorenes, a boron–tin exchange reaction was utilized. Instead of using BX_3_ (X = Cl or Br or I), PhBCl_2_ was used directly (Scheme 3, path e).^65^ The drawback of this methodology is that only sterically relatively unencumbered groups (*e.g.*, phenyl and pentafluorophenyl) can be used;^61^*e.g.*, MesBCl_2_ (Mes = mesityl group) is unsuitable for this methodology. By changing the boron source from PhBCl_2_ to dimethoxy aryl borates, and subsequent reaction with dilithiobiphenyl, 9-aryl-9-borafluorenes can be synthesized (Scheme 3, path f).^66,67^ There are two advantages to this approach: (1) dimethoxy aryl borates can tolerate coordinating solvents and are much more stable than their corresponding aryldihaloboranes, which makes the work up much easier; and (2) bulkier aryl groups (*e.g.*, 2,4,6-triisopropylphenyl (Tip)) can be used. The third approach which can assemble 9-aryl-9-borafluorenes in one step was reported by Marder and co-workers,^45,68^ in which more stable aryltrifluoroborate salts were used as the boron source (Scheme 3, path g).^69–74^ The downside of this approach is the comparably low yields. Besides these three widely applied approaches, which can assemble 9-aryl-9-borafluorenes in one step, the Wehmschulte group synthesized two unsymmetric 9-borafluorenes (2a and 2b, Scheme 3, path h) in one step by using H_2_ClB·SMe_2_ as the boron source.^75,76^ The formation of the borole ring takes place *via* a facile intramolecular C–H activation process, but the requirement of bulky terphenyl precursors limits its further application.

**Scheme 4:**
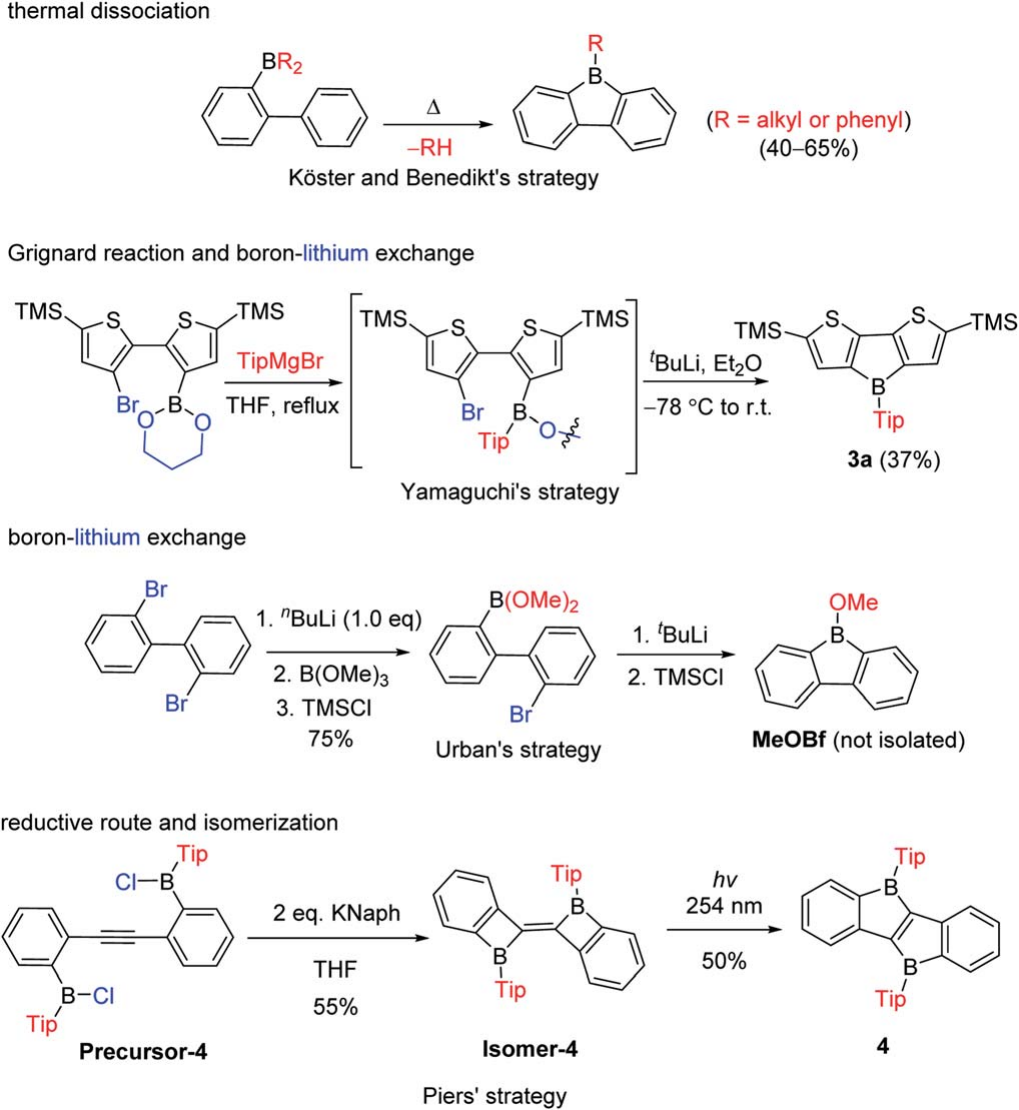
Stepwise substitution reactions to synthesize fused boroles.

The third approach is a stepwise substitution reaction at boron, which was first reported in 1963 by Köster and Benedikt (Scheme 4).^55^ Thus, 9-alkyl-9-borafluorenes can be synthesized from *B*,*B*-dialkyl-2-biphenylborane intermediates by thermal dissociation of one alkyl-group at 180–200 °C. The compound 9-phenyl-9-borafluorene (PhBf) can also be synthesized from *B*,*B*-diphenyl-2-biphenylborane in the same way, but the temperature needs to be increased to 280–300 °C. The harsh conditions in this approach limit its further application. In 2011, the Yamaguchi group applied boronic esters as the boron source for the synthesis of heteroarene-fused boroles.^77^ Using the dithiophene-fused borole (3a) as an example, the boronic ester was introduced at the bithiophene *via* boron–lithium exchange in the first step, then the protecting group at the boron was introduced with a Grignard reagent. Finally, an intramolecular cyclization reaction completed the synthesis of 3a. More recently, Urban and co-workers applied a similar strategy, but used an intramolecular cyclization in the second step, obtaining 9-methoxy-9-borafluorene (MeOBf),^78^ a potential intermediate for synthesizing other 9-substituted-9-borafluorenes. In 2012, the Piers group used a reductive route with a haloborane (Precursor-4, obtained by three stepwise boron–metal exchange reactions) while attempting to synthesize the diborole 4 (Scheme 4, bottom). Instead, they initially obtained an isomer of 4 (Isomer-4) which, under irradiation with UV light (254 nm), isomerized to the desired diborole 4.^79^ Subsequently, the same group reported another, more efficient, thermal route to the diborole 4 from Isomer-4.^80^

## Stability of 9-borafluorenes

The advantage of incorporating a boron atom into a 5-membered diene-ring is the enhancement of electron accepting ability and Lewis acidity, but at the same time, stability is sacrificed. By fusing two phenyl rings onto a borole, the stability is greatly enhanced. The stability of the resulting 9-borafluorenes depends largely on the *exo*-substituent at boron. In this section, we compare the stability of different 9-borafluorenes.

**Scheme 5:**
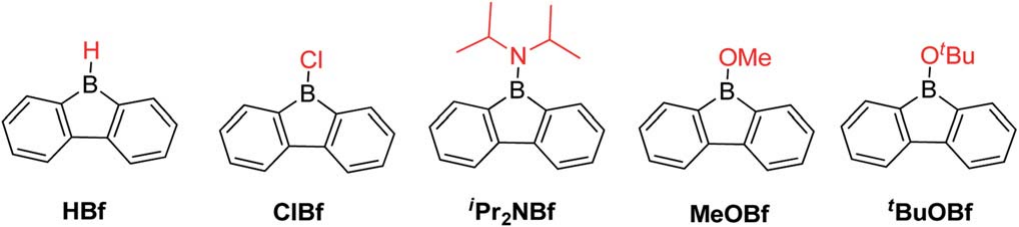
Non-aryl group substituted 9-borafluorenes.

When the *exo*-substituent is not a bulky aryl group, 9-borafluorenes remain highly reactive (Scheme 5). In fact, HBf in an unsymmetric dimer in solution and, after some time, it forms oligomers *via* a ring-opening mechanism even in dry and deoxygenated C_6_D_6_.^55,81–84^ClBf^85–87^ and ^i^Pr_2_NBf both show more than 50% decomposition within 1 hour in solution when exposed to the atmosphere.^88^*^t^*BuOBf is much more stable and exhibits less than 10% decomposition after 1 hour in CDCl_3_ in air. This can be attributed to the steric demand of the *tert*-butyl group as the less bulky derivative MeOBf^59^ is as sensitive to air/moisture as ^i^Pr_2_NBf and ClBf.^88^

**Scheme 6:**
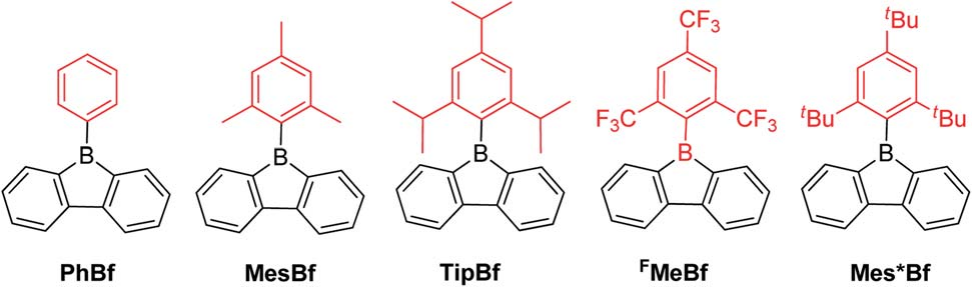
9-Aryl-9-borafluorenes.

**Fig. 2:**
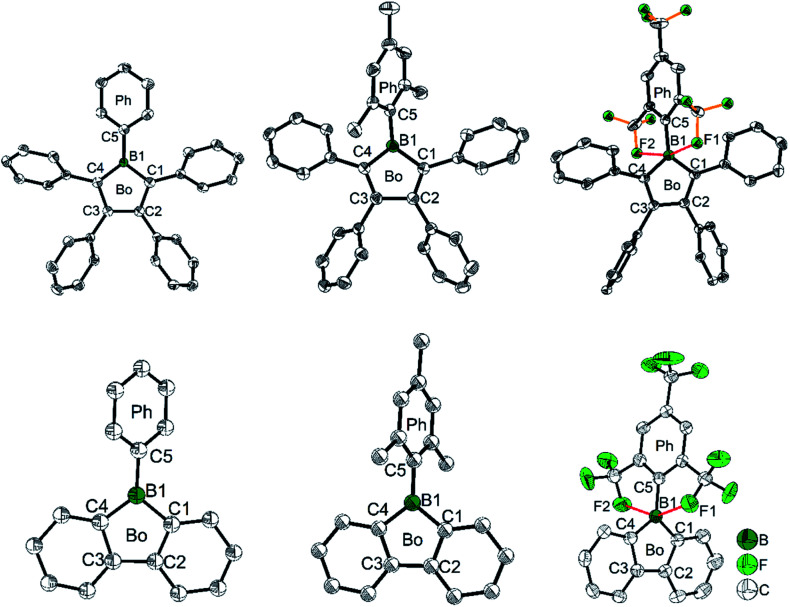
Molecular structures of PhB (top, left), MesB (top, middle), ^F^MesB (top, right), PhBf (bottom, left), MesBf (bottom, middle) and ^F^MesBf (bottom, right). For PhBF and ^F^MesBf, only one of the two symmetry independent molecules is shown. Hydrogen atoms and the minor component of disordered CF_3_ groups of ^F^MesB are omitted for clarity. Red dashed lines in ^F^MesB and ^F^MesBf indicate short B–F contacts. ‘Bo’ and ‘Ph’ denote the planes of the borole and the *exo*-aryl groups, respectively.

An aryl group as the *exo*-substituent at 9-borafluorene increases the stability drastically (Scheme 6). PhBf and MesBf decompose only slowly in the air.^54^PhBf nonetheless retains high reactivity, *e.g.*, azides can insert into one of the B–C bonds of PhBf to generate 9,10-*B*,*N*-phenanthrenes.^62,89^ The reaction of PhBf with 1,2-dipolar substrates leads to the formation of the corresponding ring expansion adducts.^90,91^ By employing Tip or ^F^Mes as the protecting group at the boron atom of 9-borafluorenes, their stability is greatly enhanced, and both derivatives can be purified by column chromatography, even in air.^66,88^ Stability tests indicate that TipBf shows *ca.* 15% decomposition in solution in air after 24 hours and ^F^MesBf shows only *ca.* 5% decomposition under the same conditions. Considering that the trifluoromethyl group is less bulky than the isopropyl group,^92^ this observation initially seems counterintuitive. In fact, the higher stability of ^F^MesBf is likely due to the stabilizing interaction of the vacant p_*z*_-orbital of boron by lone pairs of the fluorine atoms of the two *ortho*-CF_3_ groups of the *exo*-^F^Mes. This σ-donation was confirmed by the short B⋯F distances (2.682(6) and 2.577(5) Å) observed in the solid state (Fig. 2), which are much shorter than the sum of the van der Waals radii for boron and fluorine (3.39 Å).^93^ The σ-donation from the fluorine atom(s) of *ortho*-CF_3_ group(s) to the vacant p_*z*_-orbital of boron was also observed in other boranes^94–102^ and boroles.^45,68^ The 2,4,6-tri-*tert*-butylphenyl (Mes*) group is the bulkiest substituent among these protecting groups and, thus, provides the most stable 9-borafluorenes.^103^ Compared to TipBf, which still exhibits reactivity towards the small F^−^ anion and can be applied as a F^−^ sensor, Mes*Bf is inert to F^−^. This demonstrates the superior stability of Mes*Bf.

To gain a deeper understanding of the relation between the structure and the stability, selected bond parameters of PhBf,^104^MesBf,^105^ and ^F^MesBf^88^ derived from single crystal X-ray studies are listed in Table 1 and the molecular structures are depicted in Fig. 2. The free non-fused boroles, PhB,^42^ 1-mesityl-2,3,4,5-tetraphenylborole (MesB)^44^ and 1-(2,4,6-tritrifluoromethylphenyl)-2,3,4,5-tetraphenylborole (^F^MesB),^45^ are included for comparison. Compared to PhB, the C–B bond lengths of the other five compounds are significantly longer. The short C–B bond lengths of PhB are a result of the strong p_π_(B)–π* conjugation. The C1–C2 and C3–C4 bonds in MesB (1.356(2) and 1.351(2) Å) and ^F^MesB (1.359(3) and 1.358(3) Å) are obviously double bonds, but in the other four compounds, these bonds are significantly longer. In contrast, the remaining inner-borole C2–C3 bond is quite long in MesB (1.537(2)) and ^F^MesB (1.526(3)) compared to those in PhB and the three fused boroles. So, similar to PhB, the C_ring_–C_ring_ distances in fused boroles are indicative of some electron delocalization, which is in stark contrast to typical non-fused boroles such as MesB, resulting in significant differences in the properties of fused *vs.* typical non-fused boroles. The ∠C1–B1–C4 angles of the six compounds shown in Fig. 2 are quite similar. The torsion angles between the *exo*-aryl ring bonded to boron and the borole core are 32.71(5)° (PhB), 68.7(1)° (MesB), 82.4(3)° (^F^MesB), 32.8° and 39.2° (PhBf), 84.1° (MesBf), 80.9(12)° and 82.2 (2)° (^F^MesBf) (the unit cell of PhBf and ^F^MesBf exhibit two distinct molecules), respectively. Apparently, the bulkier the *exo*-aryl group is, the larger the torsion angle becomes, resulting in more efficient kinetic protection.

**Table 1:** Selected bond lengths (Å) and angles (°) of PhB, MesB, ^F^MesB, PhBf, MesBf and ^F^MesBf. For PhBf and ^F^MesBf, only one of the two symmetry independent molecules is listed

	PhB	MesB	^F^MesB	PhBf	MesBf	^F^MesBf
B1–C1	1.526(2)	1.586(2)	1.571(3)	1.573(9)	1.566(2)	1.550(6)
B1–C4	1.539(2)	1.575(2)	1.576(3)	1.59(1)	1.567(2)	1.556(5)
B1–C5	1.516(2)	1.560(2)	1.580(3)	1.58(1)	1.565(2)	1.584(5)
C1–C2	1.428(2)	1.356(2)	1.359(3)	1.431(9)	1.416(2)	1.421(5)
C3–C4	1.426(2)	1.351(2)	1.358(3)	1.421(9)	1.415(2)	1.411(5)
C2–C3	1.470(2)	1.537(2)	1.526(3)	1.51(1)	1.485(2)	1.485(5)
B1–F1			2.385(3)			2.682(6)
B1–F2			2.556(5)			2.576(5)
∠C1–B1–C4	105.4(1)	105.2(1)	106.19(17)	104.2(5)	103.7(1)	105.1(3)
∠Bo–Ph	32.71(5)	68.7(1)	82.4(3)	39.2	84.1	82.2(2)

## 9-Borafluorenes with a fluorinated backbone

**Scheme 7:**
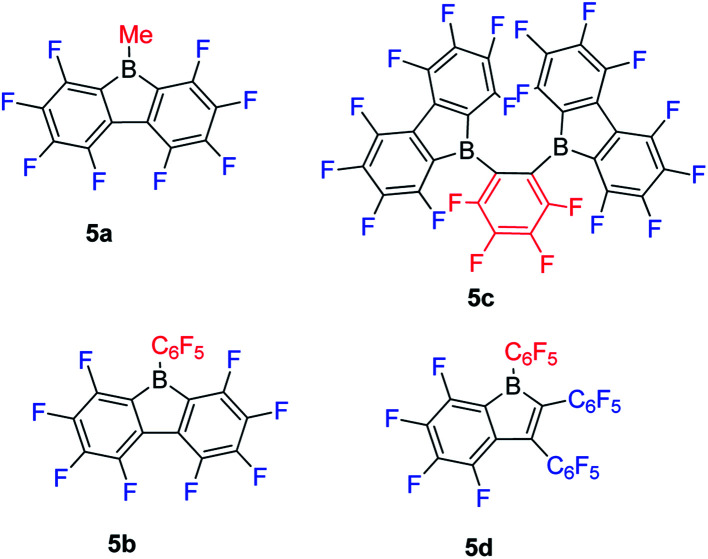
9-Borafluorenes with a fluorinated backbone.

Inspired by the wide application of B(C_6_F_5_)_3_,^106–108^ and the fact that 9-borafluorenes are more Lewis acidic than their corresponding boranes, Piers and co-workers synthesized a series of 9-borafluorenes with fluorinated backbones (Scheme 7). Compounds 5a and 5b are pale yellow or orange solids which exhibit lowest energy absorption maxima at 398 nm and 440 nm, respectively, in hexane.^61^ To explore the effect of two fluorinated 9-borafluorene centers in a molecular framework on the Lewis acidity, 5c was synthesized.^109^ In 5c, two fluorinated 9-borafluorenes are situated *ortho* to one another, forming a chelating bidentate Lewis acid. Compound 5c is a deep orange solid with a lowest energy absorption maximum at 425 nm (*ε* = 590 M^−1^ cm^−1^) in hexane, comparable to that of 5b, indicating that the two chromophores of the fluorinated 9-borafluorenes are not coupled. To the best of our knowledge, 5d is the only example of mono-aryl fused borole.^110^ Compound 5d is a red solid with its lowest energy absorption maximum at 465 nm (*ε* = 900 M^−1^ cm^−1^ in toluene), and is readily soluble in most solvents.^111^

Compared with the corresponding fluorinated perfluoroboranes (MeB(C_6_F_5_)_2_, B(C_6_F_5_)_3_ and C_6_F_4_-1,2-[B(C_6_F_5_)_2_]_2_),^109,112–114^ fluorinated 9-borafluorenes show stronger Lewis acidities, as demonstrated by Lewis base competition reactions, the Childs method,^115^ and semiempirical MNDO calculations.^116^ Apparently, compared with the corresponding fluorinated triarylboranes, the loss of two fluorine atoms is compensated by the antiaromaticity and strain of the 5-membered borole ring. Weak Lewis bases (LBs), *e.g.*, THF and CH_3_CN, both bind to these four fluorinated 9-borafluorenes. After introduction of a Lewis base, the orange solutions of 5a and 5c, or lime green solution of 5b, become colorless,^117^ and the red solution of 5d turns pale yellow.^111^ This color change is due to the interruption of p_π_(B)–π* conjugation upon coordination of the Lewis base to the boron center, which results in a higher LUMO energy.^66^

In a CH_3_CN competition reaction between 5b and B(C_6_F_5_)_3_·CH_3_CN at 25 °C, an equilibrium constant of *ca.* 1.3 was found indicating a preference for formation of 5b·CH_3_CN *vs.* B(C_6_F_5_)_3_·CH_3_CN.^117^ In another competition experiment, with the bulkier THF as the base (in a ratio of 1 : 1 : 1 for 5b, B(C_6_F_5_)_3_ and THF in *d*_8_-toluene), only the 5b·THF adduct was observed by NMR spectroscopy. Applying the Childs method, 5a and 5b have a relative Lewis acidity value of 0.58 ± 0.02 and 0.70 ± 0.02, respectively, which is only slightly higher than that of the corresponding MeB(C_6_F_5_)_2_ (0.56 ± 0.02) and B(C_6_F_5_)_3_ (0.68 ± 0.02) obtained by Piers.^61^ For the smaller Lewis base CH_3_CN, 5b and B(C_6_F_5_)_3_ show comparable Lewis acidities, but for the larger Lewis base THF, 5b shows a much stronger Lewis acidity than B(C_6_F_5_)_3_. Based on these results, the authors concluded that the relative Lewis acidities of 5b and B(C_6_F_5_)_3_ are determined by steric factors, rather than the antiaromaticity of 5b.

Addition of Cp_2_Zr(CH_3_)_2_ to 5a or 5b, leads to Me^−^ abstraction, and the corresponding ion pairs are formed rapidly.^61^ Both of them are remarkably more stable than their corresponding borane ion pairs in toluene (the ion pairs formed from MeB(C_6_F_5_)_2_ and Cp_2_Zr(CH_3_)_2_ can exchange a C_6_F_5_ group from MeB(C_6_F_5_)_2_ with a methyl group from Cp_2_Zr(CH_3_)_2_, resulting in Me_2_B(C_6_F_5_) and Cp_2_Zr(CH_3_)(C_6_F_5_) under similar conditions).^118^ The combination 5a/Cp_2_Zr(CH_3_)_2_ and 5b/Cp_2_Zr(CH_3_)_2_ are more active and stable than their corresponding borane/Cp_2_Zr(CH_3_)_2_ ion pairs as activators for olefin polymerization. To investigate further the coordination chemistry of 5a and 5b, [Cp*Al]_4_ was used.^65^ Surprisingly, only the thermally robust η^1^ Lewis acid–base adduct was observed. Thus, the fragment of Cp*Al behaves only as a Lewis base rather than as a two-electron reducing agent. The reaction of Cp*Al with the less Lewis acidic PhBf also provides the η^1^ Lewis acid–base adduct. Alternative routes to η^5^ 9-borafluorene aluminum complexes *via* reaction of PhBfLi_2_ with Cp*AlCl_2_(THF) were also unsuccessful. The reaction of 5b with L^*t*Bu^ScR_2_ (L^*t*Bu^ = ((Ar)NC(^*t*^Bu)CHC(R)N(^*t*^Bu)), Ar = 2,6-^i^Pr-C_6_H_3_) produced the corresponding contact ion pairs, the structures of which were thoroughly investigated both in solution and the solid state.^119^

**Scheme 8:**
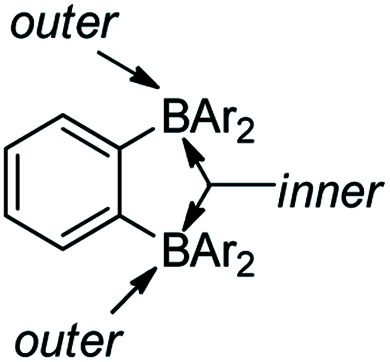
A description of facial coordination modes of *ortho*-phenylene-bridged diboranes.

*Ortho*-phenylene-bridged diboranes are interesting compounds and can be applied as co-initiators for olefin polymerizations.^120^ Depending on the binding position of the Lewis base, *ortho*-phenylene-bridged diboranes (and diborole 5c) can adopt inner or outer facial coordination modes (Scheme 8).^112–114,121^ By adding a neutral Lewis base, *e.g.*, CH_3_CN or THF, coordination to the less sterically encumbered outer face of 5c was observed exclusively.^109^ This is in contrast to the corresponding diborane C_6_F_4_-1,2-[B(C_6_F_5_)_2_]_2_, to which CH_3_CN coordinates to the inner face. The authors suggested that this is likely a result of less strain in the outer coordination mode caused by pyramidalization of the boron center in 5c; it may also be a result of the more rigid adjacent borafluorene's steric interaction which prevents the inner coordination mode in 5c. The reaction of 5c with PhCMe_2_X (X = Cl, OMe or N_3_) gives thermally stable and isolable ion pairs which feature a weakly coordinating anion (WCA). The application of these ion pairs as initiators for isobutene polymerization were studied and the results show that the combination of C_6_F_4_-1,2-[B(C_6_F_5_)_2_]_2_ with PhCMe_2_X is more suitable than 5c with PhCMe_2_X.^122^

**Fig. 3:**
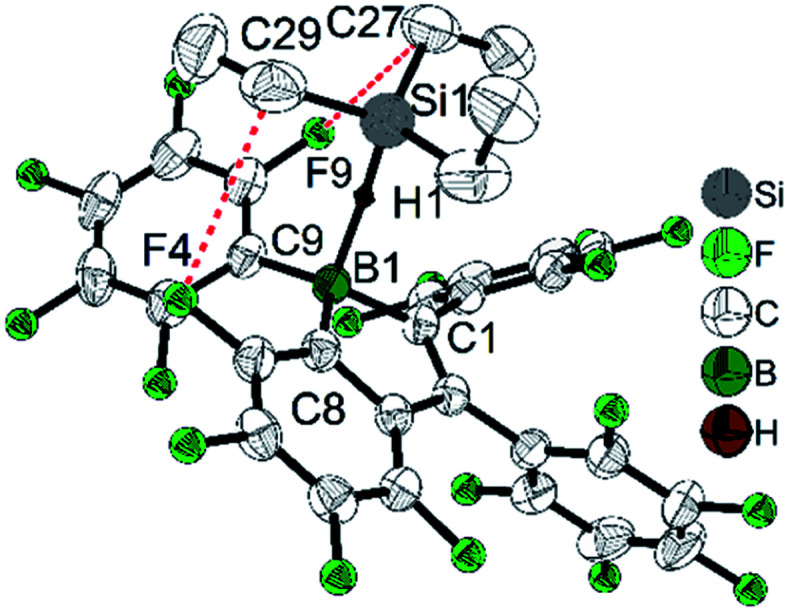
Structure of the adduct between 5d and Et_3_SiH. Only hydrogen H1 is shown, whereas other hydrogen atoms are omitted for clarity. Red dashed lines indicate the close non-bonded contacts. Selected bond lengths (Å) and angles (°): B1–C1 1.616(3), B1–C8 1.608(3), B1–C9 1.605(3), B1–H1 1.46(2), Si1–H1 1.51(2), C27–F9 3.138(3), C29–F4 3.262(2), ∠B1–H1–Si1 157, sum of ∠C–B–C 344.3(2).

While perfluoropentaphenylborole reacts rapidly and irreversibly with dihydrogen (H_2_),^123,124^5b is inert to H_2_ under various conditions. Therefore, 5d, which is a structural hybrid of 5b and perfluoropentaphenylborole, was designed and synthesized.^110^ The reaction between 5d and H_2_ was investigated experimentally and by theoretical calculations. Compound 5d reacts reversibly with H_2_, but side reactions occur resulting in only limited turnover numbers of this metal-free H_2_ activation reaction. Compound 5d has a comparable Lewis acidity to that of perfluoropentaphenylborole but exhibits a much better solubility than perfluoropentaphenylborole in non-coordinating solvents. Due to the better solubility of 5d, a low temperature experiment between 5d and Et_3_SiH was possible.^111^ The borole–silane complex formation in *d*_8_-toluene was studied by variable-temperature NMR spectroscopy. The trends of the Si–H coupling constant and the infrared stretching frequency of the Si–H bond as a function of temperature, and the molecular structure of the complex determined by X-ray diffraction (Fig. 3), clearly prove that an interaction exists between the boron atom and the silicon atom through the Si–H bond. These direct observations thus confirmed the previously proposed mechanism, *i.e.*, that perfluoroarylboranes catalyze the hydrosilylation of C=C, C=N and C=O bonds *via* borane activation of the Si–H bond, not *via* a classical Lewis acid/base adduct process.^125^

## Donor–acceptor 9-borafluorenes

**Scheme 9:**
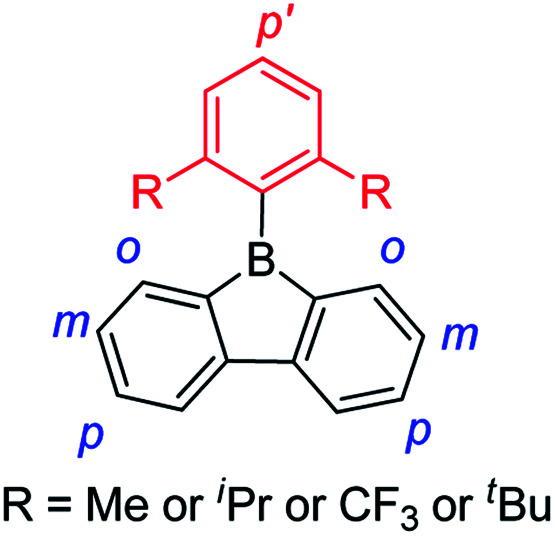
A depiction of the different positions on a 9-borafluorene to which functional groups are attached.

The 9-borafluorenes exhibit a weakly allowed lowest energy absorption which extends into the visible region. This absorption was attributed to the low-lying LUMO which originates from the p_π_–π* conjugation through the vacant p_*z*_ orbital of boron.^126^ By incorporating electron donating group(s) or electron withdrawing group(s) at different positions, the photophysical properties can be modified (Scheme 9). In this section, the 9-borafluorenes are classified according to their functional groups at different positions.

**Scheme 10:**
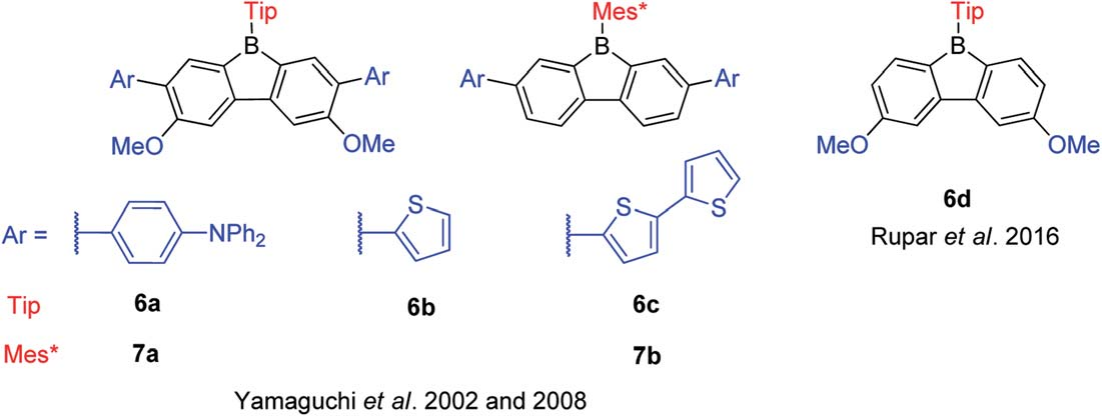
9-Borafluorenes with donors incorporated at the biphenyl core.

Pioneered by Yamaguchi and co-workers in 2002, three functionalized TipBfs (6a–6c) with donors (methoxy and amine groups or methoxy and thiophene groups) at the *m*- and *p*-positions were reported (Scheme 10).^66^ Compared with the non-functionalized TipBf, both the absorption and emission of these functionalized TipBfs are red shifted and the quantum yields decrease (Table 2). The red shifted absorption and emission of donor-functionalized TipBfs were attributed to intramolecular charge transfer (ICT). Addition of F^−^ (or coordinating solvents) leads to a blue shift of both the absorption and emission of these functionalized TipBfs and, the quantum yields dramatically increase to *ca.* 0.5–0.9. Thus, these functionalized TipBfs can be applied as F^−^ sensor. In contrast to tri(9-anthryl)borane, which loses its fluorescence properties after coordination with F^−^ and was labeled as a “turn off” sensors,^1^ due to the increase of the emission intensity after adding F^−^, these functionalized 9-borafluorenes were termed fluorescence “turn on” sensors.

**Table 2:** Photophysical data for (hetero)arene-fused boroles

Compound	Solvent	*λ*_abs_/nm (log(*ε*))	*λ*_em_/nm	*Φ*_F_
1a^68^	Hexane	400 (2.48)	521	0.37
1b^68^	Hexane	386 (2.60)	510	0.30
1c^68^	Hexane	396 (2.48)	627	0.03
3a^77^	CH_2_Cl_2_	552 (3.05)	a	a
3b^77^	CH_2_Cl_2_	469 (2.93)	a	a
3c^77^	CH_2_Cl_2_	600 (3.04)	a	a
3d^105^	CH_2_Cl_2_	479 (3.09)	b	b
3e^105^	CH_2_Cl_2_	468 (3.06)	b	b
3f^105^	CH_2_Cl_2_	474 (3.05)	b	b
5a^61^	Hexane	398 (−)	b	b
5b^61^	Hexane	440 (−)	b	b
5c^109^	Hexane	425 (2.77)	b	b
5d^110^	Hexane	465 (2.95)	b	b
TipBf^66^	THF	410 (2.39)	514	0.09
6a^66^	THF	480 (3.08)	561	0.03
6b^66^	THF	488 (2.95)	550	0.041
6c^66^	THF	504 (3.51)	576	0.022
6d^88^	CH_2_Cl_2_	398 (2.80)	499	0.10
Mes*Bf^103^	THF	397 (2.42)	501	0.35
7a^103^	THF	470 (4.06)	608	0.24
7b^103^	THF	457 (3.80)	600	0.48
8a^127^	Cyclohexane	430 (3.28)	529	0.21
8b^127^	Cyclohexane	430 (3.27)	527	0.36
8c^127^	Cyclohexane	451 (4.03)	567	0.13
9a^128^	Cyclohexane	380 (3.75)	519	0.55
9b^128^	Cyclohexane	411 (3.81)	513	0.42
[10a]_4_ (ref. 129)	THF	322 (4.70)	495	0.12
10b^129^	THF	375 (2.95)	520	0.27

a Non-emissive.

b Not reported.

Six years later, the same group synthesized another two 9-Mes*-borafluorenes (7a and 7b) with donors (amine or thiophene groups) at the *m*-positions (Scheme 10).^103^ Compared with the non-functionalized Mes*Bf, the molar extinction coefficients are much higher and a red shift was observed in both absorption and emission. Compared withtheir corresponding 9-Tip-9-borafluorenes, the fluorescence quantum yields of 9-Mes*-9-borafluorenes are higher, which is most likely due to the restricted rotation of the bulky Mes* group. The bulky Mes* group also leads to enhanced stability of these 9-Mes*-9-borafluorenes, which paves the way for their application as accepting units in organic (opto)electronics.

In 2016, Rupar and co-workers synthesized 6d with two methoxy donors at the *p*-positions (Scheme 10). The lowest energy absorption and emission peak of 6d appear in the same range as that of TipBf, and the quantum yields are also the same. The similar photophysical properties of these two compounds may be due to the weak donating ability of the methoxy groups.^88^

**Scheme 11:**
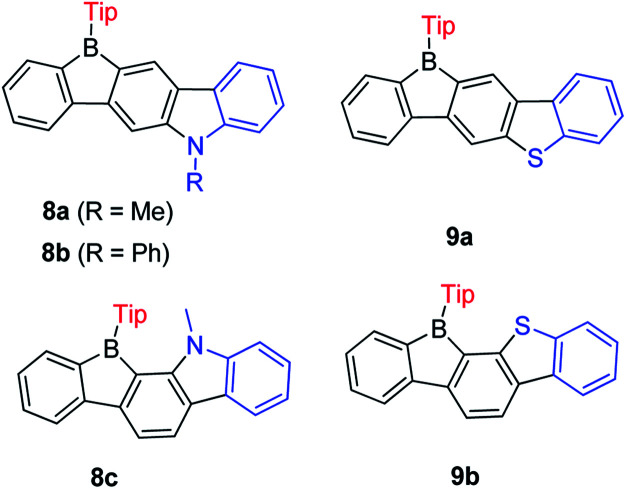
Ladder-type *B*,*N*-bridged and *B*,*S*-bridged *p*-terphenyls.

Encouraged by the wide application of carbazole as a donating group and 9-borafluorene as an accepting group, the Zhao group synthesized three ladder-type *B*,*N*-bridged *p*-terphenyls, with indole fused at the *p*-, *m*-positions (8a and 8b) or *o*-, *m*-positions (8c) on one side of the borafluorene (Scheme 11).^127^ Later, the same group replaced the indole with benzothiophene and reported another two ladder-type *B*,*S*-bridged *p*-terphenyls (9a and 9b).^128^ In these ladder-type boroles, the products of fusing at the *p*-, *m*-positions (8a, 8b and 9a), are tolerant to air and moisture, but fusing at the *o*-, *m*-positions (8c and 9b) leads to products that show slow decomposition in dilute solution in air (no specific solvent is mentioned), which is probably caused by steric congestion. Both absorption and emission show negligible solvatochromism for these ladder-type boroles which indicates only a small polarity change between the ground state and the excited state. No difference of absorption and emission was observed between *N*-methyl 8a and *N*-phenyl 8b, which may be due to the large torsion angle between the phenyl group and pyrrole. Compared to 8a, 8c shows a red shift in both absorption and emission which was attributed to the lower LUMO energy as evidenced by computational studies. Compared with carbazole-fused 9-borafluorenes, the benzothiophene fused 9-borafluorenes show only slight hypochromism of both absorption and emission but double the quantum yields. In addition to the broad use of 9-borafluorenes as F^−^ sensors, 9a was applied as a Hg^2+^ sensor because of its high affinity for Hg^2+^ due to the S atom. Additionally, these ladder-type boroles exhibit considerable potential for application as bipolar electron-transporting materials.

**Scheme 12:**
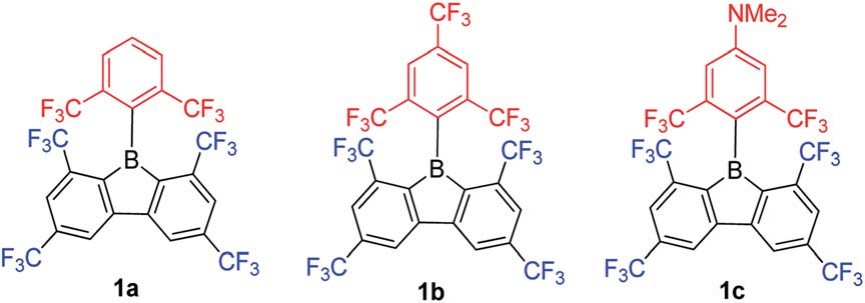
9-Borafluorenes with four CF_3_ groups at the biphenyl core.

In contrast to the incorporation of donating groups at the 9-borafluorene core, more recently, Marder and co-workers reported compound 1a with four inductively withdrawing CF_3_ groups attached to the *o*- and *p*-positions at the biphenyl main core (Scheme 12).^68^ To investigate the effect of substitution at the *p*′-position in 1a, 1b with a CF_3_ electron withdrawing group at the *p*′-position and 1c with an NMe_2_ electron donating group at the *p*′-position were also synthesized. Although examples appear in patents,^130^1c is the only example of a 3-coordinate 9-borafluorene which incorporated a donor at the *exo*-aryl to have been reported in a paper.^68^

Due to the strong electron withdrawing ability of the four CF_3_ groups at the 9-borafluorene core, the electron accepting ability of boron was greatly enhanced, as evidenced by cyclic voltammetry (see below in the Electrochemistry section). With the extra protection of two CF_3_ groups at the *o*-positions, 1a and 1b are more stable than ^F^MesBf. Surprisingly, although 1c has a strong donor at the *p*′-position, its absorption appears in the same region as those of 1a and 1b (this could also be caused by the weak absorption of 1c in the lower energy region), but the emission shows a large red shift (1a: *λ*_em_ = 510 nm; 1b: *λ*_em_ = 521 nm; 1c: *λ*_em_ = 627 nm in hexane). Both 1a (*τ*_F_ = 151 ns) and 1b (*τ*_F_ = 224 ns) exhibit very long fluorescence lifetimes in hexane, but behave differently; 1c exhibits two radiative processes (*τ*_p_ = 9.2 ns and *τ*_d_ = 1.6 μs), the latter resulting from thermally activated delayed fluorescence (TADF). Compound 1c is the first example of a borafluorene to exhibit TADF, but the rather low quantum yield (*Φ*_F_ = 0.03 in hexane) limits its further application. In contrast to the low quantum yield of 1c in hexane, 1a (*Φ*_F_ = 0.30) and 1b (*Φ*_F_ = 0.37) exhibit relatively high quantum yields. Theoretical studies indicate that the LUMO of 1c is located on the biphenyl core with a large contribution from the boron atom, whereas the HOMO is located on the *exo*-aryl moiety. Thus, the HOMO to LUMO transition is an ICT process with a small overlap coefficient (*Λ*) which also fits the requirement for TADF.

## Heteroarene-fused boroles

**Scheme 13:**
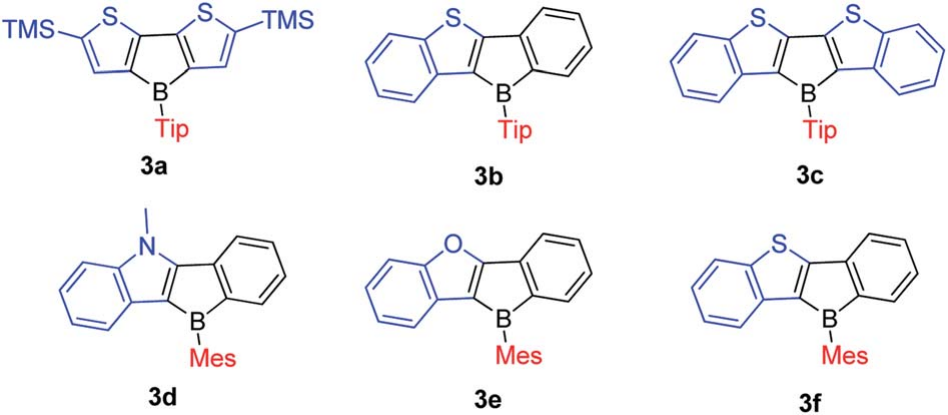
Electron-rich heteroarene-fused boroles.

In 2011, the Yamaguchi group fused electron-rich thiophene(s) onto boroles by stepwise substitution reactions and synthesized 3a–3c (Scheme 13).^77^ Surprisingly, these three Tip-protected thiophene-fused boroles are air- and moisture-sensitive. Considering that TipBf is stable enough to be purified in air, this instability is in opposition to an expectation that applying electron-rich thiophene would decrease the Lewis acidity of boron to form more stable compounds. The antiaromaticity of the borole rings was evaluated by DFT calculations of the nucleus-independent chemical shifts (NICS) values (Table 3).

**Table 3:** NICS(1)_*ZZ*_ (ppm) values for the 5-membered borole rings of (hetero)arene-fused boroles

	NICS(1)_*ZZ*_		NICS(1)_*ZZ*_		NICS(1)_*ZZ*_
1c^68^	+20.0	1a^68^	+20.2	1b^68^	+20.7
4 (ref. 79)	+21.8	MesBf^105^	+23.0	8c^127^	+24.2
TipBf^77^	+24.5	8a^127^	+24.8	8b^127^	+25.4
3e^105^	+27.6	MesB^105^	+28.3	TipB^77^	+29.4
3b^77^	+30.1	3f^105^	+30.4	3d^105^	+31.7
3a^77^	+40.3	3c^77^	+45.3		

The NICS(1)_*ZZ*_ (ppm) values increase in the order TipBf < TipB < 3b < 3a < 3c. Thus, the biphenyl-fused borole, TipBf exhibits less antiaromatic character whereas the antiaromaticity of the electron-rich thiophene-fused boroles is enhanced, and is even higher than that of the non-fused “free” borole (1-Tip-1-borole, TipB), as suggested by the NICS(1)_*ZZ*_ values. This result is also opposite to the conventional understanding that fusing electron-rich aromatic arenes decreases the antiaromaticity of the 5-membered borole ring.^131,132^

**Fig. 4:**
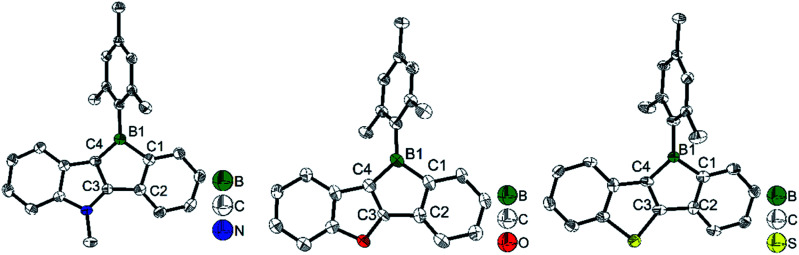
Molecular structures of 3d (left), 3e (middle), and 3f (right). Selected bond lengths (Å): 3d: B1–C1 1.613(3), C1–C2 1.419(3), C2–C3 1.471(3), C3–C4 1.394(3), C4–B1 1.528(3). 3e: B1–C1 1.612(4), C1–C2 1.408(4), C2–C3 1.490(3), C3–C4 1.347(3), C4–B1 1.549(4). 3f: B1–C1 1.595(5), C1–C2 1.400(5), C2–C3 1.495(5), C3–C4 1.375(5), C4–B1 1.554(5).

To study what governs the antiaromaticity and Lewis acidity of heteroarene-fused boroles, another three heteroarene-fused boroles, 3d–3f were synthesized (Scheme 13). NICS calculations were conducted with the geometries derived from the crystal structures of these heteroarene-fused boroles (Fig. 4). The conclusion reached was that the smaller extent of bond alternation in the 5-membered borole ring in heteroarene-fused boroles is responsible for the high degree of antiaromaticity. Theoretical and experimental studies suggest that the LUMO energy of these heteroarene-fused boroles are relevant to the antiaromaticity, which also linearly correlates with their Lewis acidities.^105^

**Scheme 14:**
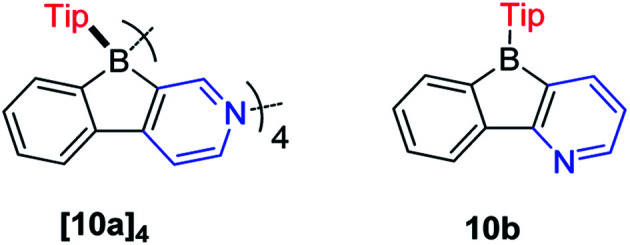
Electron-deficient pyridyl-fused boroles.

**Fig. 5:**
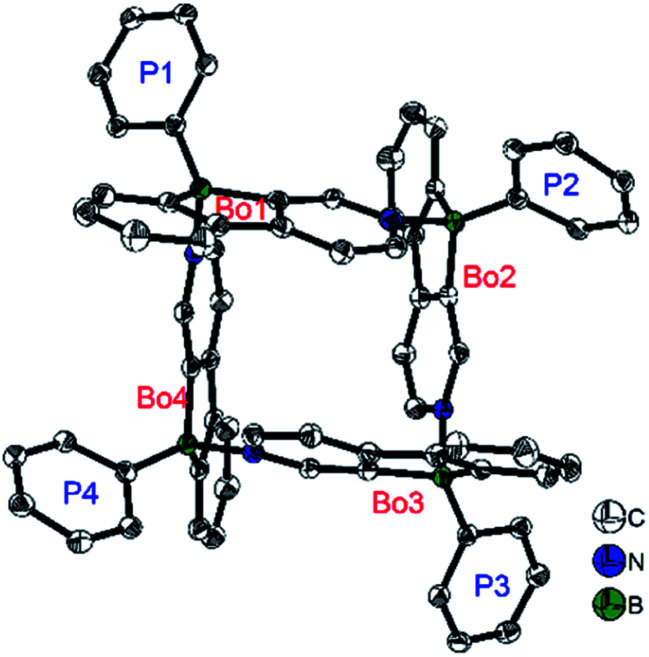
Molecular structure of [10a]_4_. H atoms, isopropyl groups, and C_6_D_6_ solvent molecules are omitted for clarity. ‘Bo’ and ‘P’ denote the planes of the phenylpyridyl-fused borole and the Tip phenyl groups, respectively. Selected bond lengths (Å) and angles (°): B_Bo1_–N_Bo4_ 1.655(2), B_Bo2_–N_Bo1_ 1.652(2), B_Bo3_–N_Bo2_ 1.644(2), B_Bo4_–N_Bo3_ 1.644(2), B_Bo1_–B_Bo2_ 5.420(2), B_Bo2_–B_Bo3_ 5.365(2), B_Bo3_–B_Bo4_ 5.407(2), B_Bo4_–B_Bo1_ 5.402(2), ∠Bo1–Bo2 89.70(2), ∠Bo2–Bo3 81.78(2), ∠Bo3–Bo4 89.22(2), ∠Bo4–Bo1 78.44(2), ∠Bo1–Bo3 33.29(3), ∠Bo2–Bo4 33.21(2).

In contrast to the Yamaguchi group's fused boroles with electron-rich heteroarene(s), more recently, Marder and co-workers^129^ switched to the electron-poor pyridine to synthesize phenylpyridyl-fused boroles (Scheme 14). Using 4-phenylpyridine to prepare a fused borole, [10a]_4_ was obtained as a white solid and adopts a unique coordination mode, forming a tetramer with a central cavity in both the solid state (X-ray diffraction, Fig. 5) and solution (^1^H diffusion-ordered spectroscopy (^1^H DOSY)). The coordination mode of [10a]_4_ is similar to that of dimethyl(3-pyridyl)borane and diethyl(3-pyridyl)borane.^133–135^ The B–N bond lengths of [10a]_4_ (1.644(2)–1.655(2) Å) are comparable to those of pentaphenylborole·2,6-lutidine (1.6567(3) Å)^136^ and sterically hindered dibenzoborole·pyridine (1.638(3) Å);^75^ however, in contrast to both pentaphenylborole·2,6-lutidine and the sterically hindered dibenzoborole·pyridine which dissociate in solution at room temperature, [10a]_4_ persists as a tetramer in C_6_D_6_ even at 50 °C (^1^H DOSY). By switching 4-phenylpyridine to 2-phenylpyridine, 10b was prepared and isolated as a light yellow solid. The boron center of 10b is 3-coordinate in solution but 4-coordinate in the solid state, as evident from solid-state ^11^B{H} RSHE/MAS NMR measurements. The difference is ascribed to the steric protection of the pyridine nitrogen by the attached phenyl group at the 2-position.

Due to the inherent electron withdrawing properties of pyridine, the electron accepting ability of 10b is enhanced (see Electrochemical section below). The lowest energy absorption maximum of [10a]_4_ appears at 322 nm in hexane, which is blue shifted compared to those of 10b (375 nm) and other 3-coordinate 9-borafluorenes. Compared with other Tip-protected 9-borafluorenes, 10b exhibits a relatively high quantum yield (0.34 in hexane) in solution and shows an interesting dual fluorescence property. Two lifetimes are observed at the same emission wavelength of 520 nm. The authors suggested that the dual fluorescence in solution is caused by an equilibrium between the free 3-coordinate 10b and a weak intermolecular coordination adduct of 10b. This hypothesis was further supported by lifetime measurements at different concentrations, low temperature excitation spectra, low temperature ^1^H NMR spectra and lifetime measurements upon addition of DMAP to a solution of 10b to simulate the 4-coordinate 10b species. Thus, this dual fluorescence is different from dual fluorescence induced by B–N dissociation in the excited state.^137^ Interestingly, the ratios of the relative percentage of the two lifetimes shows a linear relationship with temperature.

## Intramolecular dative bond in 9-borafluorenes

**Scheme 15:**
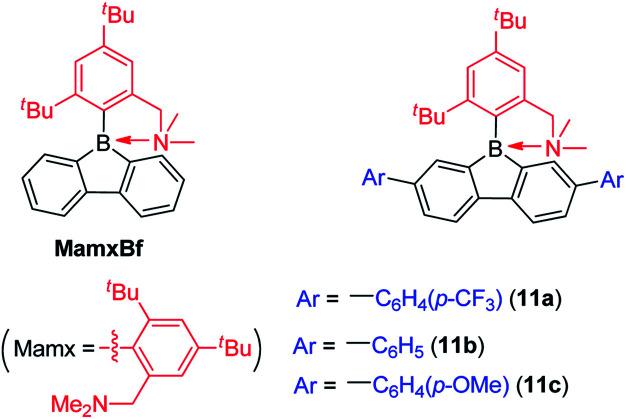
9-Borafluorenes with Mamx as the *exo*-aryl group.

Instead of using bulky Tip or Mes* as the protecting group at boron, Chujo and co-workers used the Mamx ligand (Mamx = 2,4-di-*tert*-butyl-6-[(dimethylamino)methyl]phenyl) as the steric protecting group at boron in 9-borafluorenes (Scheme 15).^138^ The X-ray crystal structure of MamxBf indicates that the nitrogen atom coordinates to the boron atom with a B–N bond length of 1.712 Å. The ^11^B NMR spectrum shows a peak at 5.96 ppm, which is in the typical range for 4-coordinate boron. With the double protection of steric hindrance and nitrogen atom coordination to boron, MamxBf is stable to moisture and can be purified in air. The lowest energy absorption of MamxBf is at *ca.* 280 nm and it exhibits a weak emission at *ca.* 360 nm. Interestingly, by addition of B(C_6_F_5_)_3_ to a benzene solution of MamxBf, phosphorescence (*τ*_p_ = 8.95 μs (69%)) with a peak at 597 nm was observed at room temperature, which the authors suggest is caused by triplet exciplexes. Theoretical analysis for the excited state of MamxBf suggests that this robust B–N coordination in the ground state is cleaved in the S_1_ state. This B–N bond cleavage in the excited state is also suggested to be responsible for the weak emission of MamxBf.

By incorporating electron withdrawing or electron donating groups at the biphenyl core, the energy of 9-Mamx-9-borafluorenes are modulated and emission from bond-cleavage-induced intramolecular charge transfer (BICT) was realized. Compound 11a has two electron withdrawing trifluoromethylphenyl groups at the biphenyl core and it shows a single emission with a peak at 373 nm, which is similar to that of MamxBf. Changing the electron withdrawing trifluoromethylphenyl groups to electron-neutral phenyl groups or electron donating methoxyphenyl groups in 11b and 11c, respectively, result in similar shaped dual emissions (*ca.* 330 nm and *ca.* 520 nm, respectively), which is in contrast to the single emission of 11a and MamxBf (Scheme 15). The authors concluded that by incorporation of electron donating groups at the biphenyl core, the boron atom exhibits a more negative charge, and a BICT process thus occurs which results in dual emission. Theoretical calculations further support the BICT transition. The short wavelength emission was assigned to a locally-excited (LE) emission from a π–π* transition and the long wavelength emission was assigned to the BICT transition. The BICT emission is highly sensitive to the solvent viscosity and, thus, 11c can be applied as a ratiometric sensor.^139^

**Scheme 16:**
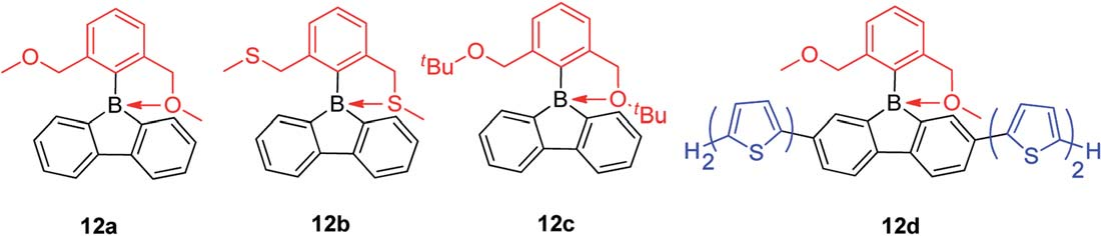
Intramolecular O→B or S→B dative bond in 9-borafluorenes.

By exchanging the strongly donating dimethylamine group with the weakly donating methoxy group, Rupar and co-workers synthesized 12a (Scheme 16)^140^ which is a colorless powder that is air-stable in the solid state and solution. The absorption maximum appears at 284 nm and the emission maximum appears at 536 nm with a long lifetime (*τ*_F_ = 122 ns) in CH_2_Cl_2_. This is an extraordinary large Stokes shift (16 500 cm^−1^) for a small molecule; in fact, it is the largest Stoke shifts ever reported.^141^ This large Stokes shift is caused by the photodissociation of the B–O dative bond in the excited state, which is further supported by theoretical studies. By changing the methoxy groups to methylthio groups or *tert*-butoxy groups, 12b and 12c were synthesized, respectively, which show nearly identical structural and optical properties to that of 12a. By incorporation of two bithiophene groups as donors at the biphenyl core of 12a, the photophysical properties change significantly. The lowest energy absorption of 12d red shifts to 408 nm and the compound exhibits dual emission with peaks at 446 (*τ*_F_ = 0.5 ns) and 639 nm (*τ*_F_ = 4.38 ns). DFT calculations indicate that two stable structures are present in the excited state: in one, the B–O bond remains intact (4-coordinate excited state) and in the other one, the B–O bond dissociates (3-coordinate excited state). The shorter wavelength emission exhibits the shorter lifetime and is assigned to the emission from the 4-coordinate S_1_ state. The long wavelength emission exhibits the longer lifetime and is assigned to the 3-coordinate S_1_ state.

**Scheme 17:**
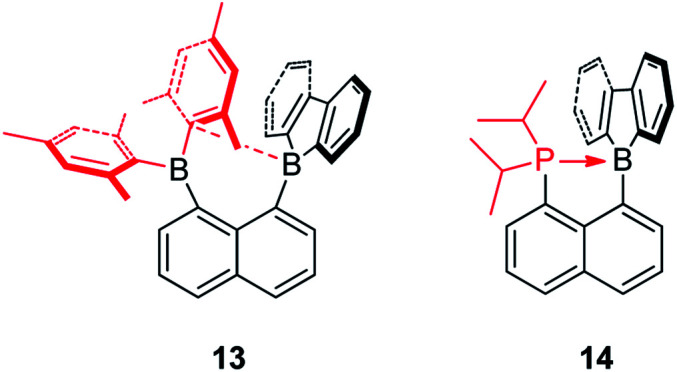
Intramolecular C→B or P→B dative bonds in 9-borafluorenes.

The Gabbaï group synthesized a diborane (13) with a BMes_2_ group and a 9-borafluorene group at the 1- and 8-positions of a naphthalene, respectively (Scheme 17).^142^ Interestingly, an interaction occurs between the boron atom of the 9-borafluorene and one of the Mes groups. This interaction was confirmed by a short B–C distance (2.730(3) Å) between the boron atom of 9-borafluorene and the carbon atom of the Mes group which is connected to boron. Due to this interaction, the boron atom of the 9-borafluorene is slightly pyramidalized. By changing the BMes_2_ group to a diisopropylphosphino group, Bourissou and co-workers synthesized the naphthyl-protected 9-borafluorene (14)^143^ which is only stable under an inert atmosphere, but is much more stable than 9-(2-diisopropylphosphinophenyl)-9-borafluorene.^144,145^ The ^11^B NMR signal appears at −8.5 ppm, confirming the presence of a P–B dative bond. The short P–B distance (2.011(2) Å) and the significant pyramidalization (*∑*_C–B–C_ = 338.45(5)°) of the boron confirmed the strong P → B interaction. This strong P → B interaction, even with fairly bulky substituents on the phosphine, indicates the flexibility of the system. In addition to the above mentioned intramolecular dative bonds in 9-borafluorenes, another interesting topic, namely intramolecular B–B dative bonds (one and two electron σ-bonds) in 9-borafluorenes which can be formed by one or two electron reductions, is discussed in the “Chemical reduction of fused boroles” section of this review (see below).

## 9-Borafluorene-based main chain polymers

**Scheme 18:**
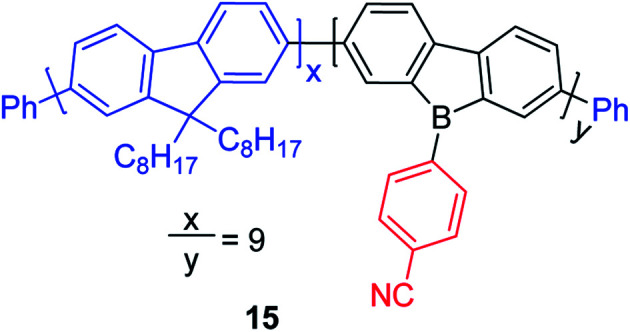
Polymer of polyfluorenes with in-chain 9-borafluorenes.

By incorporating 3-coordinate boron atoms into the main chain of conjugated polymer systems, the π-systems are extended compared to the corresponding monomers, leading to different optical properties.^22^ It could be envisaged that incorporation of more electron-deficient 9-borafluorenes into polymers will lead to interesting properties.^146^ In 2008, Scherf and co-workers reported a co-polymer incorporating polyfluorenes and 9-borafluorenes in the main chain, and applied it as an anion sensor (Scheme 18).^147^ Interestingly, the *para*-cyanophenyl group surprisingly stabilizes the 9-borafluorene, supposedly providing good environmental stability. In contrast to the bulky Tip or ^F^Mes groups, *para*-cyanophenyl is an “unprotected” phenyl group. Unfortunately, changing the *para*-cyanophenyl group to other “unprotected” phenyl groups provided unstable 9-borafluorenes. Recently, Rupar and co-workers tried to reinvestigate this compound, but although different techniques were applied, they could not reproduce the reported results.^88^

**Scheme 19:**
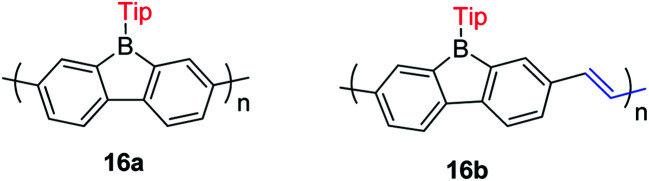
9-Tip-9-borafluorene-based polymers.

The Rupar group adopted Yamamoto or Stille coupling reactions to synthesize two 9-Tip-9-borafluorenes based polymers (Scheme 19).^148^ Compared with their monomeric precursor, a red shift of the absorption and emission was observed which can be ascribed to the extended conjugation in the polymers. Quantum yields of 0.50 (16a) and 0.28 (16b) in solution were obtained. 16a and 16b have a much smaller optical bandgap (calculated from the onset of lowest energy absorption) than polyfluorenes or polycarbazoles, which is mainly attributed to the lower LUMO energies of borafluorenes, and their lower LUMO energies were confirmed by measurements of their electrochemical reduction potentials and further supported by calculations. In solution, 16a and 16b are suitable F^−^ sensors. In a film, 16a can also be applied as a sensor for gaseous NH_3_.

**Scheme 20:**
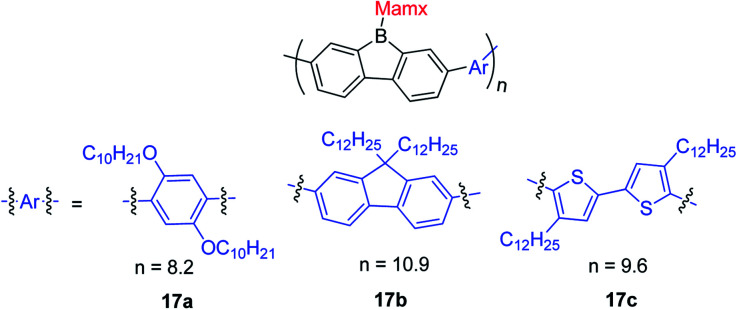
9-Mamx-9-borafluorene-based polymers.

Chujo and co-workers prepared three 9-Mamx-9-borafluorene-based conjugated polymers by Suzuki–Miyaura cross-coupling reactions (Scheme 20).^149^ These polymers are atmospherically stable and contain 4-coordinate boron. Compared with the corresponding gallafluorene polymers, the borafluorene polymers show stronger electron accepting abilities and lower LUMO energies.

## Electrochemistry

In this section, selected fused boroles are chosen for comparison of their electron accepting abilities as determined by electrochemical measurements. Although many fused boroles can be reduced twice, here only the first reduction potentials are compared. The reduction potentials for all known aryl group protected fused boroles range from −1.1 to −2.5 V (Table 4). PhBf^117^ exhibits a first reversible reduction potential at −2.16 V, which is in the same range as TipBf (−2.11 V (ref. 77) and −2.31 V (ref. 88) were obtained by two different groups) and Mes*Bf (−2.28 V).^103^ After incorporation of donating groups (methoxy, amino, or thiophene) on the core of 9-borafluorene, the first reduction potentials of 6d,^88^7a^103^ and 7b^103^ do not change significantly, indicating that the donating groups have only a small effect on the electron accepting ability of boron in 9-borafluorenes.

**Table 4:** First and second reduction potentials of (hetero)arene-fused boroles

Compound	*E*_1/2_/V (1)	*E*_pc_/V (2)	Conditions
PhBf^117^	−2.16	—	0.1 M [^*n*^Bu_4_N][B(C_6_F_5_)_4_] in THF
TipBf^77^	−2.11	−3.05	0.1 M [^*n*^Bu_4_N][PF_6_] in THF
TipBf^88^	−2.31	—	0.1 M [^*n*^Bu_4_N][PF_6_] in THF
Mes*Bf^103^	−2.28	—	0.1 M [^*n*^Bu_4_N][ClO_4_] in THF
6d^88^	−2.36	—	0.1 M [^*n*^Bu_4_N][PF_6_] in THF
7a^103^	−2.04	−2.70 (r)	0.1 M [^*n*^Bu_4_N][ClO_4_] in THF
7b^103^	−2.19	−3.00	0.1 M [^*n*^Bu_4_N][ClO_4_] in THF
^F^MesBf^88^	−1.93	—	0.1 M [^*n*^Bu_4_N][PF_6_] in THF
10b^129^	−1.94	−2.90	0.1 M [^*n*^Bu_4_N][PF_6_] in THF
3a^88^	−1.98	−2.79	0.1 M [^*n*^Bu_4_N][PF_6_] in THF
3b^88^	−1.96	−2.89	0.1 M [^*n*^Bu_4_N][PF_6_] in THF
3c^77^	−1.72	−2.61	0.1 M [^*n*^Bu_4_N][PF_6_] in THF
3d^105^	−2.25	−3.04	0.1 M [^*n*^Bu_4_N][PF_6_] in CH_2_Cl_2_
3e^105^	−1.97	−2.85	0.1 M [^*n*^Bu_4_N][PF_6_] in CH_2_Cl_2_
3f^105^	−1.89	−2.78	0.1 M [^*n*^Bu_4_N][PF_6_] in CH_2_Cl_2_
4 (ref. 80)	−1.51	−2.42	0.1 M [^*n*^Bu_4_N][PF_6_] in THF
18 (ref. 150)	−1.49	−1.75	0.1 M [^*n*^Bu_4_N][PF_6_] in THF
1a^68^	−1.21	−2.12	0.1 M [^*n*^Bu_4_N][PF_6_] in CH_2_Cl_2_
1b^68^	−1.13	−2.04	0.1 M [^*n*^Bu_4_N][PF_6_] in CH_2_Cl_2_
1c^68^	−1.28	−2.15	0.1 M [^*n*^Bu_4_N][PF_6_] in CH_2_Cl_2_
5b·THF^117^	−2.42 (ir)	—	0.1 M [^*n*^Bu_4_N][B(C_6_F_5_)_4_] in THF
^i^Pr_2_NBf^88^	−2.95	—	0.1 M [^*n*^Bu_4_N][PF_6_] in THF

**Scheme 21:**
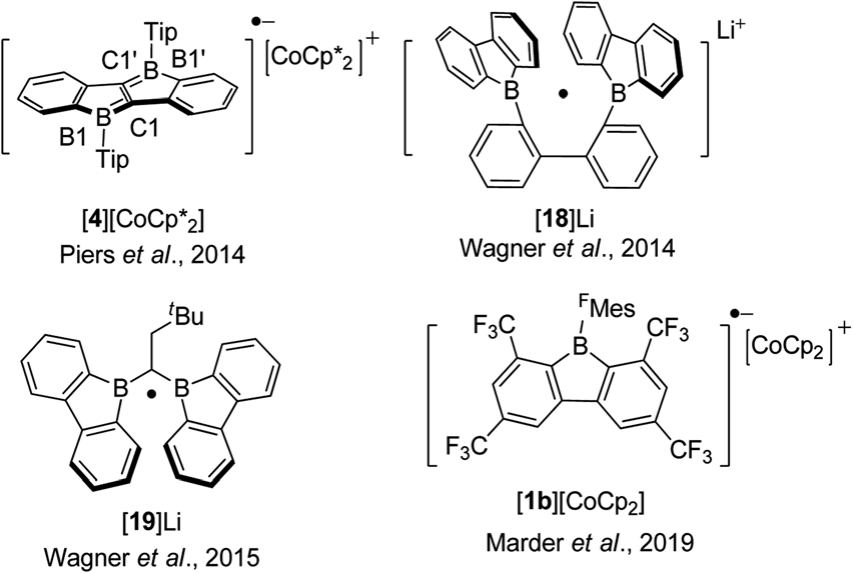
One electron reduced arene-fused boroles.

By employing the electron withdrawing ^F^Mes group as the *exo*-aryl group on a Bf, the electron accepting ability of ^F^MesBf was enhanced and the reduction potential shifts to −1.93 V.^88^ The first reduction potential of phenylpyridyl-fused borole 10b (*E*^red^_1/2_ = −1.94 V)^129^ is comparable to that of ^F^MesBf, which suggests that the effect of fusing a pyridyl group onto boroles on their reducibility is comparable to that of the *exo*-^F^Mes group in 9-borafluorenes. Benzothiophene-fused borole 3c^77^ exhibits a first reversible reduction potential of −1.72 V, which is less negative than the electron withdrawing group-functionalized ^F^MesBf and 10b. The strong electron accepting property of 3c is attributed to its enhanced antiaromaticity. Diborole 4 (ref. 80) and the biphenyl-linked diborole 18 (Scheme 21)^85,150^ exhibit much less negative half reduction potentials of −1.51 and −1.49 V, respectively. The strong electron accepting ability of 4 and 18 is due to the two boron centers being linked to a π-conjugated unit. This enhancement is also observed in triarylboranes with two or more boron centers.^100,151–153^ By incorporation of four additional CF_3_ groups at the biphenyl core, the first half reduction potentials of 1a and 1b shift to −1.21 and −1.13 V, respectively.^68^ Surprisingly, although 1a and 1b exhibit extraordinarily low reduction potentials, both are stable in air.

Attempts to use noncoordinating solvents (CH_2_Cl_2_ and α,α,α-trifluorotoluene) for the cyclic voltammetry study of perfluorinated-PhBf, 5b were unsuccessful, so THF was employed.^117^ An irreversible process with a reduction potential at −2.42 V *vs.* Fc/Fc^+^ was observed. Such a negative reduction potential, which is even more negative than that of PhBf, was not expected. The authors suggested that the reduction process actually takes place at the fluorinated aromatic framework, not at the boron center due to the formation of 5b·THF in THF. ^i^Pr_2_NBf has the most negative reversible half reduction potential of −2.95 V.^88^ The weak electron accepting ability of ^i^Pr_2_NBf is caused by the electron-rich nitrogen atom π-backbonding to the vacant p_*z*_-orbital of boron.

## Chemical reduction of fused boroles

Boroles readily accept one electron to form a radical anion or two successive electrons to give a dianion. The chemical reduction of non-fused “free” boroles and some 9-borafluorenes was reviewed by the Braunschweig group in 2011 (ref. 48) and 2016.^49^ Here we focus only on fused boroles.

One electron reduction of 9-borafluorene leads to a radical anion. In 2008, the Yamaguchi group reduced Mes*Bf with potassium in THF and the EPR signal of the reduced Mes*Bf exhibits an eleven-line signal (*g* = 2.002).^103^ According to the simulation of the spectrum, the spin density on boron is estimated to be 0.21, indicating delocalization over the biphenylene unit of the 9-borafluorene. A similar reaction was also carried out with 7b and a spin density of 0.18 on boron was estimated by simulation. The lower spin density on boron in 7b suggested that it is delocalized over the bithiophene skeleton. The Piers group synthesized the ladder-type diborole 4 which exhibits a first reversible reduction at −1.51 V. Such a small negative reduction potential makes it possible to perform a one electron reduction with bis(pentamethylcyclopentadienyl)cobalt(ii) (*E*^0^′(Cp*_2_Co) = −1.9 V).^154^ Isolated [4][CoCp*_2_] is a deep blue solid (Scheme 21).^80^ The C1–C1′ distance in [4][CoCp*_2_] of 1.410(3) Å is significantly longer than that of its neutral form for which *d*(C1–C1′) = 1.367(5) Å while *d*(B1–C1) of [4][CoCp*_2_] with 1.524(3) Å is significantly shorter compared to 1.571(4) Å for 4. A detailed inspection of the structure combined with a theoretical analysis shows that there is still a high degree of delocalization of the unpaired electron throughout the whole π system.

**Fig. 6:**
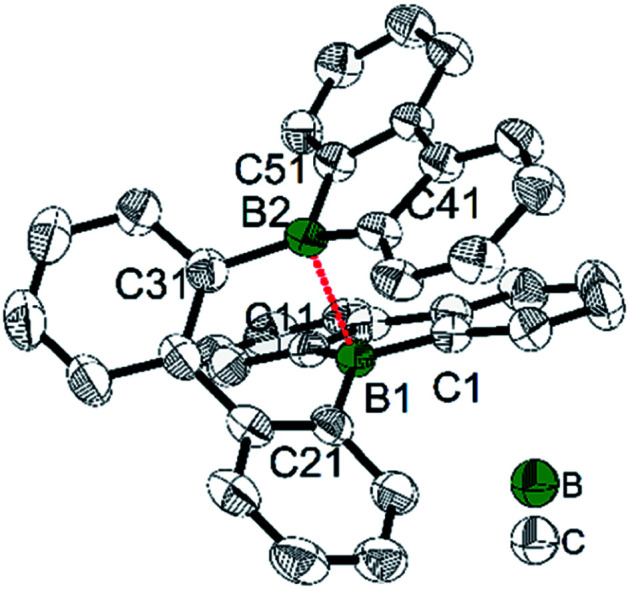
Molecular structure of [18]˙^−^. Hydrogen atoms, THF, C_10_H_8_ and the lithium cation are omitted for clarity. The dashed red line indicates the one-electron σ-bond. Selected bond lengths (Å) and angles (°): B1–B2 2.265(4), B1–C1 1.579(3), B1–C11 1.582(3), B1–C21 1.583(3), B2–C41 1.582(3), B2–C51 1.578(3), B2–C31 1.576(3), sum of ∠C–B1–C 351.6, sum of ∠C–B2–C 353.0.

In 2014, Wagner and co-workers linked two 9-borafluorenes by a biphenyl (18) and carried out the one electron reduction with lithium naphthalenide in toluene (Scheme 21),^150^ obtaining [18]Li·(THF)_4_ × 0.5C_10_H_8_ as black crystals. Single crystal X-ray diffraction shows that the distance between the two boron centers of [18]Li·(THF)_4_ × 0.5C_10_H_8_ is 2.265(4) Å, which is 0.655 Å shorter than that in its precursor 18 (2.920(6) Å) (Fig. 6),^84^ and lies between those of 18 (no B–B bond) and [HBf–HBf][K·Et_2_O]K (1.83(2) Å) or [HBf–HBf][Na·(THF)_3_]_2_ (1.822(4) Å, B–B two electron σ-bond. It is important to note that [HBf–HBf]Na_2_ can be prepared in quantitative yields and represents an extremely rare example of diborane(6) dianions).^155–158^ In [18]Li·(THF)_4_ × 0.5C_10_H_8_, only a moderate pyramidalization of the two boron centers was observed (*∑*_C–B–C_ = 351.6° and 353.0°). The EPR spectrum of [18]Li in THF exhibits a seven-line signal. The spectrum was successfully simulated, assuming the two boron nuclei to be magnetically equivalent (*a*(^11^B) = 4.8 ± 0.1 G and *a*(^10^B) = 1.6 ± 0.1 G), and the small *a*(^11^B) value strongly supports the contribution of the unpaired electron to a 2p_*z*_σ(B·B) bond. The computed SOMO and the localization of the spin-density mainly between the two boron centers further confirm the existence of a B·B one electron σ-bond. [18]Li·(THF)_4_ × 0.5C_10_H_8_ is the first crystallographic characterized compound to have a B·B one electron σ-bond.

One year later, a radical anion containing two 9-borafluorenes linked by a substituted methylene group, [19]Li, was synthesized by a comproportionation reaction between neutral 19 and its dianion, [19]Li_2_.^158^ The radical anion [Li(THF)_4_][19]·(THF)_4_ × THF was characterized by single-crystal X-ray diffraction and exhibits a B⋯B distance of 2.166(4) Å. *Via* simulation of the EPR spectrum, comparable hyperfine coupling values of [19]˙^−^ (*a*(^11^B) = 5.1 ± 0.1 G and *a*(^10^B) = 1.7 ± 0.2 G) to that of [18]˙^−^ were obtained. Based on the small *a*(^11^B) value, as well as the low degree of pyramidalization of the boron atom in [Li(THF)_4_][19]·(THF)_4_ × THF, the authors concluded that there is a strong contribution from p_*z*_ orbital on each boron to the SOMO. This is similar to the situation in [18]˙^−^, but the hyperfine coupling to protons in [19]˙^−^ suggests that the spin density is delocalized over the π-systems.

The tetrakis-CF_3_-functionalized ^F^MesBf, 1b synthesized by Marder and co-workers exhibits a first half-reduction potential at −1.13 V, and thus can be reduced by Cp_2_Co (*E*^0^′(Cp_2_Co) = −1.3 V).^154^ [1b][Cp_2_Co] was obtained as a dark purple solid with a complex EPR signal centered at *g*_iso_ = 2.004 in THF, consisting of hyperfine splitting to boron (*a*(^11^B) = 3.3 G), the fluorine atoms (*a*(^19^F) = 11.3 and 6.0 G) from the CF_3_ groups at the 9-borafluorene core, and the hydrogen atoms (*a*(^1^H) = 6.1 and 2.9 G) at the 9-borafluorene core. The relatively large proton and fluorine hyperfine couplings, in contrast to the relatively small boron hyperfine coupling, indicates that the spin density is delocalized significantly over the biphenyl core of the 9-borafluorene. Compared to the crystal structure of neutral 1b, changes in bond lengths of [1b][Cp_2_Co] were mainly observed on the 9-borafluorene core, indicating delocalization of the unpaired electron on the 9-borafluorene core, with no contribution from the *exo*-aryl ^F^Mes group.^68^

**Scheme 22:**
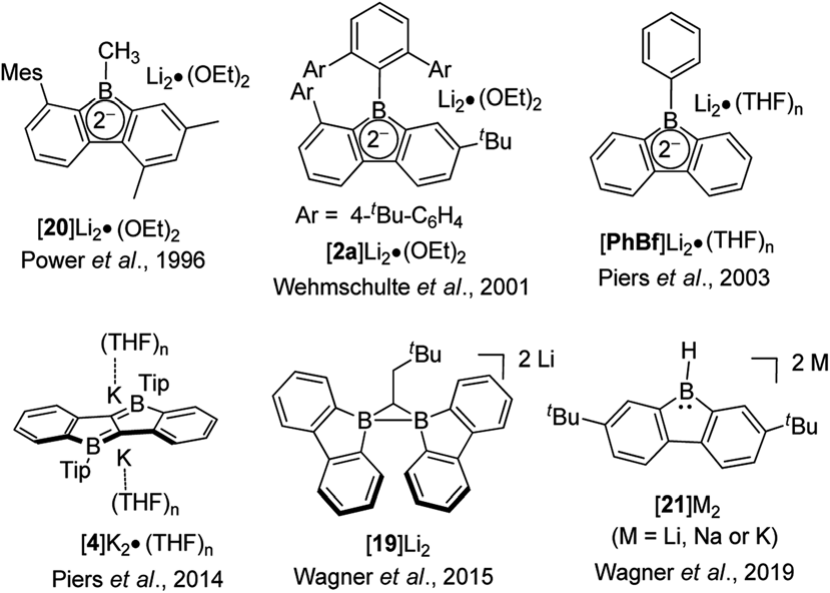
Two electrons reduced arene-fused boroles.

The discovery and study of 9-borafluorenyl dianions were reported earlier than that of the radical anions of 9-borafluorenes. In 1996, during an investigation of the reduction of sterically encumbered arylboron dihalides, the Power group isolated a bislithium-9-borafluorenyl complex [20]Li_2_·(Et_2_O)_2_ (Scheme 22),^155^ obtained directly by treatment of 2,6-Mes_2_C_6_H_3_BX_2_ (X = Br or Cl) with an excess of lithium in Et_2_O. [20]Li_2_·(Et_2_O)_2_ is a red solid with a ^11^B NMR (C_6_D_6_) chemical shift of 14.3 ppm (s, *W*_1/2_ ≈ 380 Hz). [20]Li_2_·(Et_2_O)_2_ was the first structurally characterized 9-borafluorenyl dianion, the core of which is still planar. The lithium ions are solvated by diethyl ether and adopt an η^5^-coordination to the 5-membered borole ring. Reduction of 2,6-Mes_2_C_6_H_3_BBr_2_ with lithium in benzene over 5 days and extraction with ether/hexane gave a dimer that has a structure analogous to that of [20]Li_2_·(Et_2_O)_2_. [2a]Li_2_ (ref. 75) and [PhBf]Li_2_ (ref. 65) were isolated by treatment of the corresponding 9-borafluorenes with lithium in diethyl ether or THF. [2a]Li_2_ is a deep red, almost black solid with a ^11^B NMR (C_6_D_6_) chemical shift of 13.6 ppm (s, *W*_1/2_ ≈ 430 Hz). The two lithium ions in [2a]Li_2_·(Et_2_O)_2_ are also solvated by diethyl ether and are situated almost symmetrically above and below of the 5-membered borole core. The ^11^B NMR (*d*_8_-THF) chemical shift of [PhBf]Li_2_ shows a sharp peak at 6.3 ppm. By treatment of diborole 4 with potassium naphthalenide (2 eq.) in THF, [4]K_2_ was isolated as a red solid with a ^11^B NMR (C_6_D_6_) chemical shift of 32.1 ppm. Its X-ray crystal structure indicates that the two potassium atoms are situated above and below the center of the dibenzo-fused-diborole core in a centrosymmetric arrangement.^80^ Similarly to diborole 4, compound 19 possesses two 9-borafluorene moieties and thus can easily accept two electrons.^158^ The dianion [19]Li_2_ was obtained as a red solid with a ^11^B NMR (*d*_8_-THF) chemical shift of −6.7 ppm (*h*_1/2_ = 270 Hz). The distance between the two boron atoms in [19][Li(Et_2_O)_2_][Li(Et_2_O)] is 1.906(3) Å, which is shorter than that in its radical anion ([Li(THF)_4_][19]·(THF)_4_ × THF, 2.166(4) Å) and neutral form (19, 2.534(2) Å). The ∠B–C–B angles decrease from 105.5(2)° ([19][Li(Et_2_O)_2_][Li(Et_2_O)]) to 86.9(2)° (radical anion [Li(THF)_4_][19]·(THF)_4_ × THF), and to 73.2(1)° (neutral form 19). The spectroscopic and structural parameters clearly suggest the presence of a covalent two center-two electron (2c-2e^−^) B–B bond in [19]Li_2_, which was further supported by theoretical studies.

The Wagner group also investigated the redox chemistry of 9-H-9-borafluorene, 21 (Scheme 22).^159,160^ Upon reduction, adducts arising from extensive structural rearrangement were formed and the corresponding mechanism was studied. Besides rearrangement adducts, recently, the dianion [21]^2−^ was isolated upon reduction of monomeric 21·THF.^157^ Treatment of 21 with lithium, sodium or potassium in THF led to the isolation of the respective dianions. [21]Li_2_·(THF)_3_ and [21][Na·(THF)_3_][Na·THF] were obtained as greenish-brown solids and [21]K_2_·(THF) is a brown solid. The ^11^B NMR (*d*_8_-THF) chemical shifts are 3.7 ppm (s, *W*_1/2_ ≈ 290 Hz, [21]Li_2_), 3.9 ppm (s, *W*_1/2_ ≈ 290 Hz, [21]Na_2_) and 8.0 ppm (s, *W*_1/2_ ≈ 360 Hz, 21K_2_). From a comparison of the bond lengths of the computed structure of neutral 21 and of the reduced form [21][Na·(THF)_3_][Na·THF], the authors draw the conclusion that the two added electrons are delocalized over the 9-borafluorene core, rather than being localized at the p_*z*_-orbital of boron. [21]^2−^ is the first example of a hydride ligand-stabilized 9-borafluorene anion. Due to the easy abstraction of the hydride, [21]^2−^ is a surrogate of a nucleophilic 9-borafluorene anion. The reaction of [21]^2−^ with MeI and Et_3_SiCl further proves that [21]^2−^ can be applied as a 9-borafluorene anion. Similarly, reaction of B–B dianions with MeI led to the formation of 9-methyl-borafluorenes.^161,162^ At the same time, radical reactivity of [21]^2−^ was found, *e.g.*, by the reaction of [21]^2−^ with Me_3_SnCl or (bromomethyl)cyclopropane.

## Three-coordinate borafluorenium cations

**Scheme 23:**
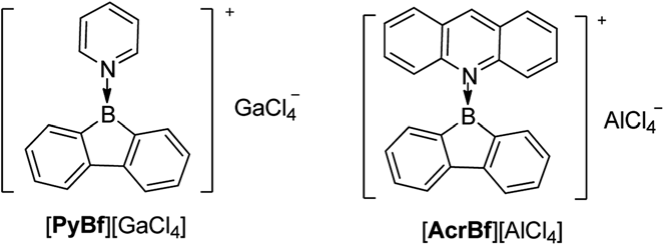
Pyridine or acridine coordinated borafluorenium cations.

Instead of adding electrons to Bfs in a reduction process, another interesting topic is extracting an anion from 3-coordinate Bf to generate a 3-coordinate borafluorenium cation. In 1985, Nöth and co-workers applied GaCl_3_ and AlCl_3_ as a Cl^−^ acceptor for the pyridine adduct of ClBf and the acridine adduct of ClBf (Scheme 23).^163^ Both [PyBf][GaCl_4_] and [AcrBf][GaCl_4_] are red solids. Due to the insolubility of [PyBf][GaCl_4_] in most solvents, the only direct proof of the formation of [PyBf][GaCl_4_] is its IR spectrum which exhibits a strong band at 373 cm^−1^. This band is in the typical range for *ν*_abs_ (GaCl_4_).^164^ The structure of [AcrBf][GaCl_4_] was confirmed by single crystal X-ray diffraction, but without further characterization or study.

**Scheme 24:**
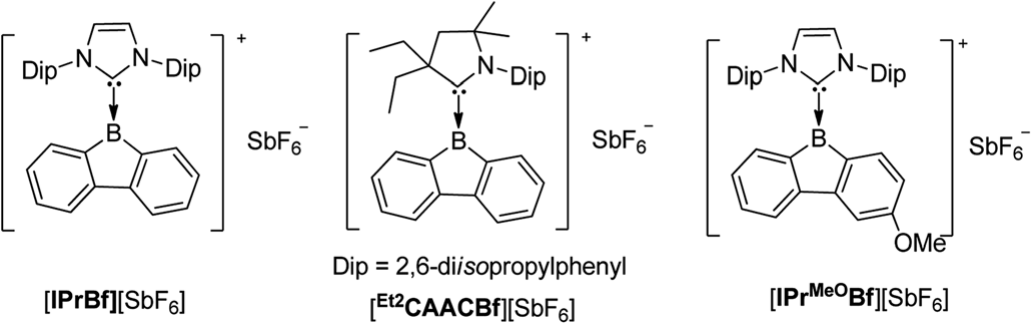
Carbene-stabilized borafluorenium cations.

Interested in the properties of borafluorenium cations, recently, Gilliard Jr and co-workers extracted a bromide ion from carbene-stabilized BrBfs with AgSbF_6_ and synthesized [IPrBf][SbF_6_] and [^Et2^CAACBf][SbF_6_] (Scheme 24).^165^ The ^11^B NMR spectra of [IPrBf][SbF_6_] and [^Et2^CAACBf][SbF_6_] show signals at 63.6 and 65.5 ppm, respectively, confirming that these two borafluorenium cations are 3-coordinate. Inspired by the decolorization upon addition of THF, they designed [IPr^MeO^Bf][SbF_6_] to tune the color.

**Scheme 25:**
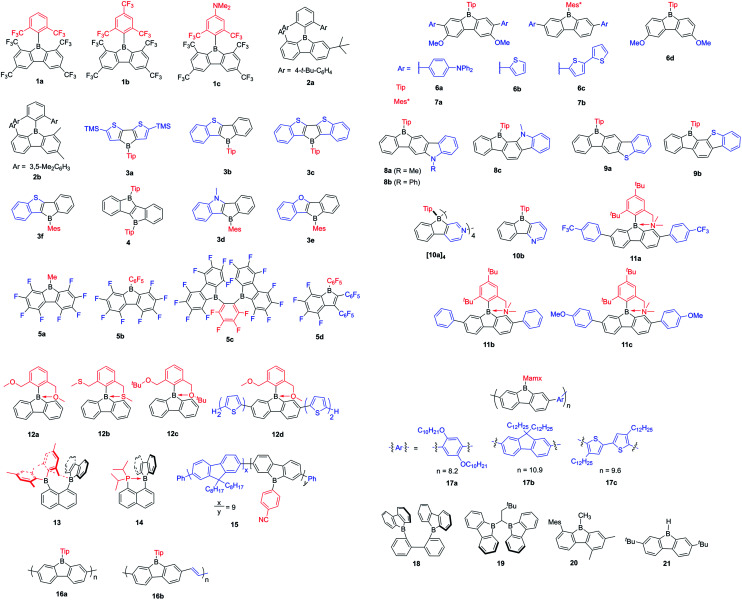
Structures of compounds with Arabic numbers.

**Fig. 7:**
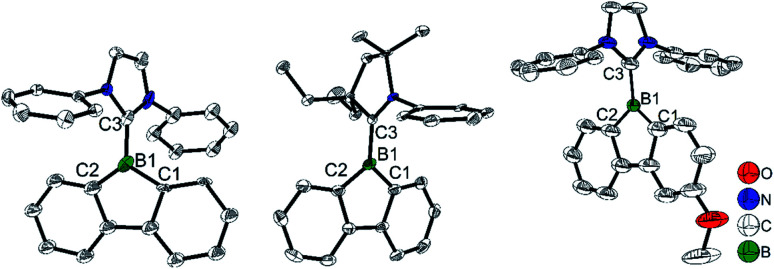
Molecular structures of [IPrBf][SbF_6_] (left), [^Et2^CAACBf][SbF_6_] (middle) and [IPr^MeO^Bf][SbF_6_] (right). H atoms, isopropyl groups and [SbF_6_]^−^ are omitted for clarity. Selected bond lengths (Å) and angles (°) [IPrBf][SbF_6_]: B1–C1 1.57(2), B1–C2 1.528(3), B1–C3 1.618(2), ∠C1–B1–C2 111.9(10). [^Et2^CAACBf][SbF_6_]: B1–C1 1.553(10), B1–C2 1.567(10), B1–C3 1.581(10), ∠C1–B1–C2 104.7(6). [IPr^MeO^Bf][SbF_6_]: B1–C1 1.509(7), B1–C2 1.602(7), B1–C3 1.586(6), C_sp^2^_–O 1.354(8), ∠C1–B1–C2 106.4(4).

The single-crystal X-ray diffraction analyses indicate that the two C–B bond lengths in the borole ring are the same for [IPrBf][SbF_6_] (C1–B1 1.615(11) and C2–B1 1.57(2) Å, Fig. 7) and [^Et2^CAACBf][SbF_6_] (C1–B1 1.553(10) and C2–B1 1.567(10) Å). However, in [IPr^MeO^Bf][SbF_6_], the C1–B1 bond (1.509(7) Å) is much shorter than the C2–B1 bond (1.602(7) Å). The authors suggested that this is caused by the conjugation of the lone pair on oxygen with the cationic boron center. Interestingly, compared to [IPrBf][SbF_6_] and [^Et2^CAACBf][SbF_6_] which exhibit a very weak absorbance in the 400–600 nm range in CH_2_Cl_2_, a strong absorbance at 430–600 nm in CH_2_Cl_2_ was found for [IPr^MeO^Bf][SbF_6_]. Another interesting finding is that [IPr^MeO^Bf][SbF_6_] shows thermochromic behavior; the red color of [IPr^MeO^Bf][SbF_6_] faded to colorless in non-coordinating solvents upon cooling. The authors suggested that this phenomenon is caused by an intermolecular O⋯B interaction of [IPr^MeO^Bf][SbF_6_] which is favored at low temperature. Attempts to grow colorless crystals of [IPr^MeO^Bf][SbF_6_] which feature an O⋯B interaction at low temperature were unsuccessful. Furthermore, to support their hypothesis, THF was added to simulate the intermolecular coordination. After addition of 50 equivalents of THF to a red CH_2_Cl_2_ solution of [IPr^MeO^Bf][SbF_6_], the solution became colorless. When this solution was heated to 40 °C, the red color was recovered. After cooling, this solution became colorless again. These phenomena further support the hypothesis of an intermolecular O⋯B interaction at low temperature.

## Conclusions and outlook

This review begins with the fundamental synthetic strategies for preparing (hetero)arene-fused boroles and the stability of different 9-substituent-9-borafluorenes, and then discusses functionalized (hetero)arene-fused boroles which can be applied as Lewis acids, activators of H_2_, fluorescent materials, electron accepting units, *etc.* For functionalized (hetero)arene-fused boroles, the chemistry of reported 9-borafluorenes is classified, and a guide for the design of novel (hetero)arene-fused boroles to achieve different properties is provided.

Compared to the corresponding triarylboranes, (hetero)arene-fused boroles exhibit an enhanced electron accepting ability, which is attributed to the antiaromaticity and strain of the 5-membered borole ring. Triarylboranes have found wide application, *e.g.*, as acceptors in TADF materials. The *exo*-aryl group of 9-aryl-9-borafluorene adopts a twisted arrangement with respect to the 9-borafluorene core and thus, by functionalization, may also generate good candidates for TADF materials. Surprisingly, thus far, only one such example was reported outside of patents. More studies on the functionalization of the *exo*-aryl moiety will be of particular interest.

Compared to non-fused “free” boroles, arene-fused boroles exhibit higher stability and potential for functionalization. Depending on the fused aryl groups, enhanced electron accepting ability and enhanced antiaromaticity, even greater than that of non-fused “free” boroles, unique coordination modes, and dual fluorescence can be realized. Heteroarene-fused boroles are interesting compounds which require further study, (*e.g.*, other electron-rich or -poor heteroarene-fused boroles), as they have many potential applications.

## Conflicts of interest

There are no conflicts to declare.

## Supplementary Material
